# Amelioration of CCl_4_ induced liver injury in swiss albino mice by antioxidant rich leaf extract of *Croton bonplandianus* Baill.

**DOI:** 10.1371/journal.pone.0196411

**Published:** 2018-04-30

**Authors:** Somit Dutta, Arnab Kumar Chakraborty, Priyankar Dey, Pallab Kar, Pokhraj Guha, Subhajit Sen, Anoop Kumar, Arnab Sen, Tapas Kumar Chaudhuri

**Affiliations:** 1 Cellular Immunology Laboratory, Department of Zoology, University of North Bengal, Siliguri, West Bengal, India; 2 Molecular Genetics Laboratory, Department of Botany, University of North Bengal,Siliguri, West Bengal, India; 3 OMICS Laboratory, Department of Biotechnology, University of North Bengal, Siliguri, West Bengal, India; 4 ANMOL Laboratory, Department of Biotechnology, University of North Bengal, Siliguri, West Bengal, India; 5 Visiting Professor, Department of Zoology, Bodoland University, Kokrajhar, Assam, India; Jadavpur University, INDIA

## Abstract

The progress in industrialization has blessed mankind with a technologically superior lifestyle but poor management of industrial waste has in turn poisoned nature. One such chemical is carbon tetra chloride (CCl_4_), which is a potent environmental toxin emitted from chemical industries and its presence in the atmosphere is increasing at an alarming rate. Presence of CCl_4_ in human body is reported to cause liver damage through free radical mediated inflammatory processes. Kupffer cells present in the liver are potentially more sensitive to oxidative stress than hepatocytes. Kuffer cells produced tumor necrosis factor-α (TNF-α) in response to reactive oxygen species (ROS), that might further cause inflammation or apoptosis. In this study hepatoprotective capacity of antioxidant rich extract of *Croton bonplandianus* Baill. (CBL) was evaluated on CCl_4_ induced acute hepatotoxicity in murine model. Hydro-methanolic extract of *C*. *bonplandianus* leaf was used for evaluation of free radical scavenging activity. Liver cells of experimental mice were damaged using CCl_4_ and subsequently hepatoprotective potential of the plant extract was evaluated using series of *in-vivo* and *in-vitro* studies. In the hepatoprotective study, silymarin was used as a positive control. Antioxidant enzymes, pro-inflammatory markers, liver enzymatic and biochemical parameters were studied to evaluate hepatoprotective activity of *Croton bonplandianus* leaf extract. Free radical scavenging activity of CBL extract was also observed in WRL-68 cell line. The phytochemicals identified by GCMS analysis were scrutinized using *in-silico* molecular docking procedure. The results showed that CBL extract have potent free radical scavenging capacity. The biochemical parameters were over expressed due to CCl_4_ administration, which were significantly normalized by CBL extract treatment. This finding was also supported by histopathological evidences showing less hepatocellularnecrosis, inflammation and fibrosis in CBL and silymarin treated group, compared to CCl_4_ group. ROS generated due to H_2_O_2_ in WRL-68 cell line were normalize in the highest group (200 μg/ml) when compared with control and negative control (CCl_4_) group. After molecular docking analysis, it was observed that the compound α-amyrin present in the leaf extract of *C*. *bonplandianus* has better potentiality to protect hepatocellular damages than the standard drug Silymarin. The present study provided supportive evidence that CBL extract possesses potent hepatoprotective capacity by ameliorating haloalkane induced liver injury in the murine model. The antioxidant and anti-inflammatory activities also affirm the same. The synergistic effects of the phytochemicals present in CBL are to be credited for all the hepatoprotective activity claimed above.

## Introduction

In aerobic system, oxygen and nitrogen are abundantly found. These molecules take part in various physiological and metabolic processes and undergo changes [1]. The molecules inturn gets transformed to unpaired moieties which are readily reactive, example singlet oxygen, superoxide, nitric oxide etc. [2]. The oxygen free radicals and nitrogen free radicals are such reactive moieties. Later it was found that apart from unpaired free radicals certain paired non-radical compounds like hydrogen peroxide, ozone and peroxynitrite are equally harmful [3]. So, the terms Reactive Oxygen Species (ROS) and Reactive Nitrogen Species (RNS) were coined to include all these reactive radical and non-radical molecules which are produced by different endogenous and exogenous sources inside living system [4]. Although, ROS and RNS have certain beneficial role like signal transduction, bodies defence mechanisms against microbes etc., and are required in trace amount inside our body. But, due to their chemical nature they helps lipid peroxidation causes DNA damage and oxidize several molecules including cellular membrane and causes injury [5]. Inspite of having a strong antioxidant mechanism (natural antioxidant in body), cell damage from ROS/ RNS is pervasive. Oxidative/nitrosative stress is an essential phenomenon associated in this regard. It occurs due to an imbalance between the production of this oxygen free radical (oxidants) and their elimination by defence mechanisms referred to as antioxidant. Similarly, nitrosative stress is also equally dangerous and arises mainly due to nitrogen free radical [6].

Hepatic cells are equally if not more vulnerable to such stresses [7]. Oxidative stress is a commonly used term that refers to a state when the cellular redox balance is altered [8]. The parenchyma and the non-parenchyma cells of liver are equally affected by oxidative stress, although both show different response. The mitochondrion microsome and perxoisome in parenchyma cells can produce ROS, which triggers the regulation of PPARA gene. The PPAR α, δ, γ are involved in kitogenesis. Although parenchyma cells consists of 70–80% of liver volume and covers bulk of the liver constitution, the non-parenchyma cells also plays a huge role on oxidative stress. The changes in composition of parenchyma cells as well as extra cellular matrix of liver due to oxidative and nitrosative stress modulates immune system. Immune and inflammatory cells get recruited at the site of injury and this activates non-parenchyma cells like hepatic stellate cells and kupffer cells [9]. There is a substantial increase in levels of growth hormone, cytokines and chemokines which leads to fibrosis and chronic liver diseases. Liver fibrosis is the gateway to several liver-related ailments and among all the reasons for liver dysfunctions, the exposure to toxic reagents and drug cannot be ignored. Carbon tetrachloride (CCl_4_) has been widely used for liver studies for long now [10].

*Croton bonplandianus* Baill. (*C*. *bonplandianus*) (Euphorbiaceae) is an exotic weed commonly found in wastelands which possess medicinal properties [11]. Leaf of *C*. *bonplandianus* is used in the treatment of skin diseases and applied traditionally in external cuts and wounds [12]. Leaf of *C*. *bonplandianus* contains diterpene resins which are reported to have antitumor activity [13]. *C*. *bonplandianus* is reported to possess potent hepatoprotective, anti-helminthic activity and also used for controlling high blood pressure [14–16]. Inspite of having diverse medicinal properties ethnobotanically, their proper pharmacognostic evidence is largely obscure. The present study aims to investigate the hepatoprotective potential of *C*. *bonplandianus* in CCl_4_ induced murine model.

## Materials and methods

### Preparation of plant extract

Leaf of *Croton bonplandianus* was collected during April and May, 2016, from the garden of medicinal plants of Department of Botany, University of North Bengal (26.7072^0^ N; 88.3558^0^ E). The plant was identified by plant taxonomist of Department of Botany, University of North Bengal. The specimen (Accession number-09870) was deposited at the Herbarium of the same department. Leaves of *C*. *bonplandianus* (CBL) were separated and washed thrice with distilled water to remove dirt and dried at 50°C for two hours to eliminate moisture. Dried leaves were then milled with a grinder (Maharani, India, Model–Sujata Dynamix). A fine powdered leaf was stored in a refrigerator at −20°C. One hundred gm of the dried powder was stirred in 1 L of 70% methanol for 10 hours. The mixture was refluxed for 2 hours in soxhlet apparatus and centrifuged at 8000 rpm for 15 minutes. Supernatant was collected and concentrated by Rotary evaporator (45°C) and finally freeze dried. Extract was stored in air-tight vessel at −20°C for further studies.

### Ethical statement

*Croton bonplandianus* (accession number-09870) was collected from the university campus area. These places are not under a National Park/Reserve Forest/Govt. protected area. All the experiments using animals were reviewed and approved by the Animal Ethical Committee of Department of Zoology, University of North Bengal (Permit No. 840/ac/04/CPCSEA, Committee for the Purpose of Control and Supervision of Experiments on Animals). The experiments with animals were performed in accordance with the legislation for the protection of animals used for scientific purposes.

### Animal maintenance

Swiss albino mice (6 male/group) were used for hepatoprotective analyses. Male Swiss albino mice were used for all the experiments. Mice (6 male/group, n = 6) were kept in polypropylene cages (Tarson, India), with paddy husk as bedding material. The experimental mice were maintained in the animal house of the Department of Zoology, University of North Bengal with sufficient food and water *ad-libitum* under a constant 12 hour dark/light cycle at an environmental temperature of 25°C.

### Determination of *in vitro* antioxidant activity

Total antioxidant, DPPH (2,2-diphenyl-1-picrylhydrazyl) radical scavenging, hydroxyl radical scavenging, superoxide radical scavenging, nitric acid radical scavenging, singlet oxygen scavenging, reducing power, Fe^2+^ chelation, peroxynitrite scavenging and hypochlorous acid scavenging activities were determined by following the previously reported methods with minor modification for the evaluation of free radical scavenging properties of CBL extract [17,18].

### Determination of erythrocyte-membrane stabilizing activity

Erythrocyte membrane stabilizing activity of CBL extract was performed by standard method as described by Dey et al. [19]. Briefly, varying concentrations of CBL extract (0–200 μg/ml) was added to the mixture of 50 mM phosphate buffer (0.5 ml; pH 7.2), distilled water (1 ml), 10% RBC suspension (0.25 ml PBS), 12 mM EDTA (100 μl), NBT (150 μl of 1% solution), and riboflavin (100 μl). The solution then kept under bright light for 30 sec and incubated for 30 min at 50°C followed by centrifugation at 1000 rpm for 10 min. The absorbance of the supernatant was measured at 562 nm and compared with the standard compound, quercetin.

### Determination of total phenolic and flavonoid content

The total phenolic and flavonoid content of CBL extract was determined using standard protocol [17]. A standard curve prepared with known quantities of gallic acid (R^2^ = 0.9468) and quercetin (R^2^ = 0.9947) were used to measure the phenolic and flavonoid content respectively.

### Detection of intracellular ROS generation

A colorimetric procedure has been used to generally quantify H_2_O_2_ by oxidation of 2’-7’ dichlorofluorescin (H_2_DCF) to 2’-7’dichlorofluorescein (DCF). Originally, DCF was thought to be specific for hydrogen peroxide, but recent evidence has shown that other ROS/RNS such as nitrate and hypochlorous acid can oxidize H_2_DCF [20, 21]. Human hepatic cell line (WRL-68) was grown on coverslip in 35mm Petri-plate culture dishes and incubated for 24 hours at 37°C with 5% CO_2_ in N-biotech incubator. Then cells were treated with different experimental concentrations of extract (50, 80, 100, 150 and 200μg/ml) with CCl_4_ and two plates were kept without treatment for control. After 23hrs of incubation, in one untreated plate H_2_O_2_ (0.03%) was added again and kept for 1hr. All plates were washed twice with PBS and fresh serum media was added with 20μM 2, 7-dichlorofluorescein diacetate and incubated for 30 min at 37°C in the CO_2_ incubator. Immediately after the incubation, cells were washed thrice with serum free media and glass slides were prepared by inverting cover slips on the slide in 20% glycerine/PBS solution. Cells were observed under LED-based fluorescence microscope, Magnus MLXi microscope. The cells were excited at 480nm using LED cassettes and emission was collected using a long pass filter. Cells were observed at10X magnification and images were captured by digital SLR Olympus camera mounted on the head for high resolution image.

### Experimental design: *In vivo* hepatoprotective activity

#### Acute toxicity study

OECD guidelines (test 423: Acute oral toxicity–Acute toxic class method; 2002) were followed to study the acute toxicity of CBL extract on animal model (OECDiLibrary, 2002). Mice were divided into different groups (n = 6) and kept on fast for overnight prior to the experiment. The plant extract was administered orally in an increasing dose upto 2000 mg/kg body weight (BW) and observed carefully for the development of clinical or toxicological symptoms at 30 min and then 2, 4, 8, 24 and 48 h. No mortality was observed in the experimental mice at 2000 mg/kg dose. Therefore, 1/40^th^, 1/20^th^ and 1/8^th^ of the maximum dose was considered for the *in vivo* studies.

#### Doses

Swiss albino mice (36 mice) were randomly divided into six groups (n = 6) and following treatments were done once per day for 21 consecutive days: Control group received normal saline; CCl_4_ group received 1:1 (v/v) CCl_4_ in olive oil; Silymarin group received 1:1 (v/v) CCl_4_ in olive oil and 100 mg/kg BW silymarin; low dose extract (CBLL) group received 1:1 (v/v) CCl_4_ in olive oil and 50 mg/kg, medium dose extract (CBLM)) group received 1:1 (v/v) CCl_4_ in olive oil and 100 mg/kg and high dose extract (CBLH) groups received 1:1 (v/v) CCl_4_ in olive oil and 250 mg/kg BW respectively.

On 22^nd^ day i.e. 24 h after the last dose, under proper anesthesia (2% ether) all the animal were sacrificed by cervical dislocation to alleviate suffering. Blood was allowed to clot for 60 min at room temperature (20°C). Then serum was separated by centrifuging at 1000 rpm for 5 min from the clotted blood. The straw colored serum was used to study *in vivo* liver marker enzymes. Liver was separated from diaphragm by cutting the falciform and coronary ligaments. The liver was washed with phosphate buffer saline to remove blood. Isolated liver was homogenized and centrifuged. After centrifugation the supernatant was collected and used for *in vivo* antioxidant enzymatic assays. Liver tissue was chopped and preserved in 10% formaldehyde solution for histological study.

#### Liver function test: *In vivo*

Serum samples from each group were used to study several liver function tests like ACP, albumin, globulin, glucose, ALP, bilirubin, cholesterol, LDH, GGT, AST, ALT, total protein, urea and urea N_2_ levels using commercially available kits (Crest Biosystems, India).

#### Estimation of peroxidase activity

Peroxidase activity was estimated by measuring the oxidation of guiacol in the liver of treated mice according to a standard method [22]. 50 mg of tissue samples were homogenized in 0.1M ice cold phosphate buffer (pH 7.0) and centrifuged at 3000 rpm for 15 min for the study of peroxidase activity. The supernatant (100 μl) was mixed with 20 mM guiacol. Time was recorded for the increase of absorbance by 0.1 at 436 nm in presence of 300 μl H_2_SO_4_ (12.3 mM).

#### Estimation of catalase activity

Catalase activity was assessed by the standard protocol of Luck [23] with some modifications, wherein degradation of substrate H_2_O_2_ by catalase in the liver tissue samples was measured. 50 mg of tissue samples were homogenized in 0.05 M of 1 ml Tris-HCl buffer (pH 7.0) and centrifuged at 10,000 rpm for 10 min at 4°C for the study of catalase activity. The supernatant was collected. In a spectrophotometric cuvette, 500 μl of 0.34 mM H_2_O_2_, 2.5 ml H_2_O and 40 μl supernatant were added and change in absorbance was noted six times at 30 sec intervals at 240 nm.

#### Estimation of reduced glutathione (GSH)

Reduced glutathione activity was measured according to the standard protocol [24]. An aliquot of 1 ml liver tissue supernatant was treated with 0.5 of Elman reagent (19.8 mg DTNB dissolved in 100 ml of 0.1% sodium nitrate). After the treatment with Elman reagent, 3 ml of phosphate buffer was added and the absorbance was measured at 412 nm.

#### Estimation of superoxide dismutase (SOD)

For the estimation of superoxide dismutase, standard method was followed with minor modifications [25]. Reaction mixture was prepared using 1 ml of 50 mM sodium carbonate, 0.4 ml of 25 μM nitroblue tetrazolium and 0.2 ml of 0.1 mM freshly prepared hydroxylamine hydrochloride. Clear supernatant of liver homogenate (0.1 ml, 1:10 w/v) was added to the reaction mixture. The changes in absorbance of the sample were recorded at 560 nm.

#### Experimental design: *In vitro*

*In vitro* hepatoprotective potentiality of *C*. *bonplandianus* extracts was studied according to the previously described standardized protocol with some modifications [10, 26, 27]. Different experimental groups of primary explant culture of mice hepatocytes were prepared in RPMI-1640 medium containing 50 U/ml penicillin, 50 U/ml streptomycin and 50 U/ml nystatin supplemented with 10% fetal bovine serum (FBS) for *in vitro* experimentation. Following treatments were done after 48 h of incubation: Control had no separate treatment; CCl_4_ group received 25 μl/ml CCl_4_; Silymarin group received 25 μl/ml CCl_4_ and 100 μg/ml silymarin; low dose extract group (CBLL) received 25 μl/ml CCl_4_ and 25 μg/ml CBL; medium dose extract group (CBLM) received 25 μl/ml CCl_4_ and 50 μg/ml CBL; high dose extract groups received 25 μl/ml CCl_4_ and 100 μg/ml CBL extract. The plates were incubated for 2 h and centrifuged at 5000 rpm for 10 min. After centrifugation culture supernatant was collected for further experiments.

#### Liver function test: *In vitro*

Culture supernatant from the experimental groups were analysed for ACP, ALP, bilirubin, LDH, AST, ALT and total protein levels using commercially available kits (Crest Biosystems, India).

#### Measurement of lipid peroxidation

Estimation of lipid peroxidation or MDA content was done using TBARS assay kit (Cayman, USA) according to the manufacturer’s instructions. Supernatant was measured at 340 nm.

#### Measurement of TNF-α

TNF-α released in culture supernatants of the experimental mice were measured using TNF-α ELISA kit (Ray Bio, USA) according to the manufacturer’s instructions. Absorbance of the sample was immediately measured after the assay at 450 nm using Bio-Rad iMark™ microplate absorbance reader.

#### Measurement of inhibition of NO

Nitric oxide level was determined using the Griess reagent method [28] with some modifications. Culture supernatants of the experimental groups were taken to quantify the NO level. Briefly, 60 μl culture supernatant from each group was mixed with 240 μl of Griess reagent [(1% sulfanilamide and 0.1% N-(1-naphthyl) ethylenediamine hydrochloride in 2.5% H_3_PO_4_)] in a 96-well plate. Then the plate was incubated for 20 min at room temperature for the development of purple azo-dye. The dye was detected at 540 nm.

#### MTT cytotoxicity assay

Carbon tetrachloride (CCl_4_) creates necrosis in hepatocytes. Therefore, MTT cytotoxicity assay was performed in six sets using EZcountTM MTT Cell Assay Kit (HiMedia) according to the manufacturer’s instructions, to examine the prevention rendered by CBL extract against CCl_4_ mediated toxicity.

### Histopathological studies

Livers were removed from the experimental mice, cut into small pieces and fixed in 10% formaldehyde solution for overnight followed by dehydration. Dehydrated tissues were embedded in paraffin. 4 μm sections were cut using microtome. Then liver sections were dewaxed in xylene, rehydrated in a series of different grades of alcohol and then washed with distilled water for 5 min. The liver sections were stained with basic stain haematoxylin for 40 sec and counterstained with acidic stain eosin for 20 sec. After proper staining the slides were observed (100X and 400X) using Nikon ECLIPS E200 microscope to identify the damages like necrosis, portal inflammation, vascular congestion, fatty infiltration, vacuolar degeneration, leukocyte infiltration, loss of structure of hepatic nodules and so forth [29,30]. Fibrosis was also observed in the CCl_4_ intoxicated group.

### GC–MS analysis

CBL extract was dissolved in n-hexane and the mixture was centrifuged thrice at 12,000 rpm for 15 min for GC-MS analyses. Supernatant was collected and used for GC–MS analysis. Agilent 5975 GCMS system (Agilent Technologies, USA) attached with HP-5 ms Capillary Column (30 m × 0.25 mm i.d. × 0.25 μm film thickness) and equipped with inert MSD triple axis mass detector condition edation trap 200°C, transfer line 280°C, electronenergy70eV (vacuum pressure-2.21e0.5 Torr) was used to identify the bio active compounds present in CBL extract. Helium was used as a carrier gas at a flow rate of 1 ml/min and 2 ml sample was injected in a split less mode. The column temperature was set at 60°C for 1 min followed by 5°C/min up to 250°C and the essential compounds in CBL were identified by the retention times and mass fragmentation patterns using Agilent Chem Station integrator and the database of National Institute of Standard and Technology (NIST) with a MS library version2011.

### Molecular docking

Proteins were chosen based on literature survey, having functional implications in hepatotoxic activity. The X-ray structures of the proteins available in the Protein Data Bank (http://www.rcsb.org) were used. Molecular docking was conducted using AutoDock Vina [31]. The receptor structures were defined as rigid, and the grid dimensions were 100, 100 and 100 for the X, Y, and Z axes for proteins having PDB ID's 1nfi (IkappaBalpha/NF-kappaB complex), 1vkx (Crystal structure of p50/p65 heterodimer), 2jod (adenylate cyclase). On the other hand for proteins with PDB ID's 1ilg (Human Pregnane X Receptor), 1n3u (oxygenase-1), 3i7h (Crystal Structure of DDB1), 7api (human alpha1-antitrypsin) grid dimension were 80, 80, 80 for X, Y and Z axes respectively. Gasteiger charges were assigned for all the compounds, and nonpolar hydrogen atoms were merged. All torsions of the ligand were allowed to rotate during docking. The value for the exhaustiveness of the search was 8. All graphic manipulations and visualizations were performed using the AutoDock Tools and ligand docking with Autodock Vina.

### Statistical analysis

All data were analysed post-blank normalization and quantitative data are reported as the mean ± SD of six measurements. Statistical analysis was performed by paired t-tests using KyPlotV5.0 (32 bit) and Graph Pad Prism V6.0. P<0.05 was considered significant. Percentage of inhibition/scavenging was calculated by the following formula: (X0-X1)/X0×100, where X0 = absorbance of control and X1 = absorbance in the presence of the samples or standard. The IC_50_ (half maximal inhibitory concentration) values were calculated by the following formula: Y = A1/(X+A1)×100, where A1 = IC_50_, Y = response (Y = 100% when X = 0), X = inhibitory concentration. Extent of dose-dependent activity was calculated by pair wise linear correlation analysis between group mean percent of inhibition Vs respective concentrations.

## Results

### *In vitro* antioxidant activity

The free radical scavenging activity of CBL in dose dependent manner and the differences in the activities compared with standard compound. Half maximal inhibitory concentration (IC_50_) of CBL and corresponding references are shown in the S1 Table.

**Fig 1:**
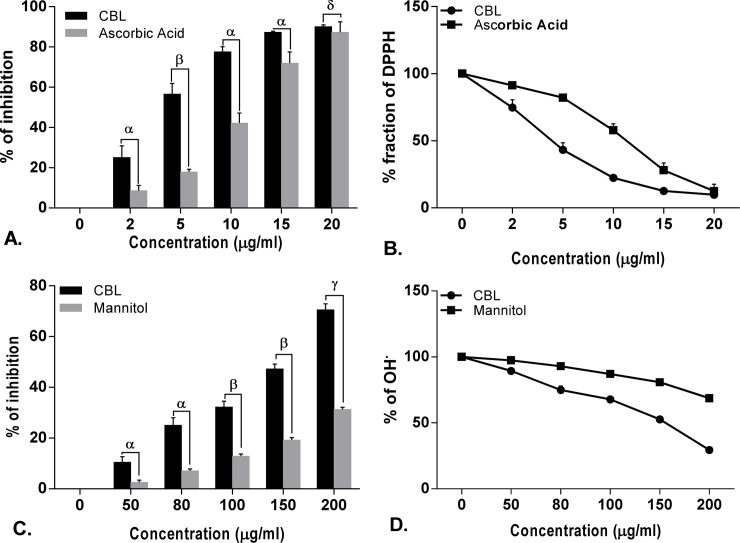
Antioxidant activity of *Croton bonplandianus*. **(A)**&**(B)** DPPH scavenging activity; **(C)** % of hydroxyl radical (OH^●^) scavenging Vs standard mannitol; **(D)** depicts remaining unneutralized OH^●^. Data expressed as mean ± S.D (n = 6). ^α^ p<0.05; ^β^ p<0.01; ^γ^ p<0.001; ^NS^-Non significant when compared with standard.

**Fig 2:**
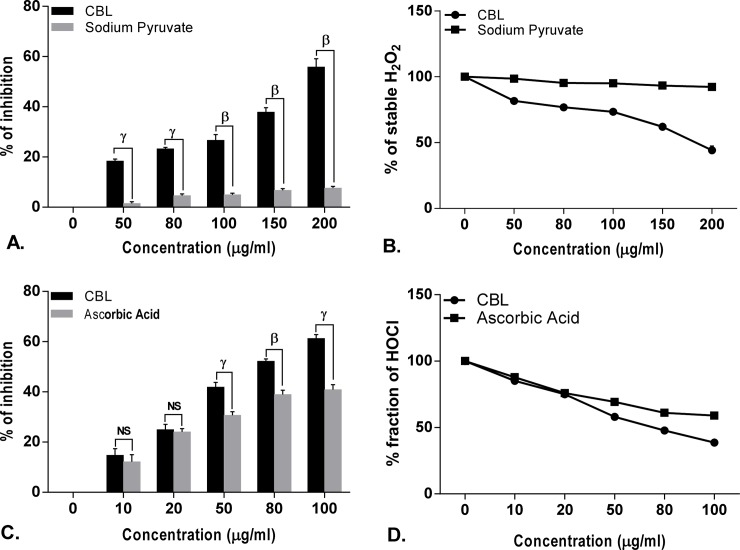
Antioxidant activity of *Croton bonplandianus*. **(A)** % of inhibition of hydrogen peroxide (H_2_O_2_)Vs standard sodium pyruvate; **(B)** depicts remaining unneutralized H_2_O_2_; **(C)** % inhibition of Hypochlorous acid (HOCl) Vs standard ascorbic acid; **(D)** depicts unneutralized HOCl radicals. Data expressed as mean ± S.D (n = 6). α p<0.05; β p<0.01; γ p<0.001; NS-Non significant when compared with standard.

**Fig 3:**
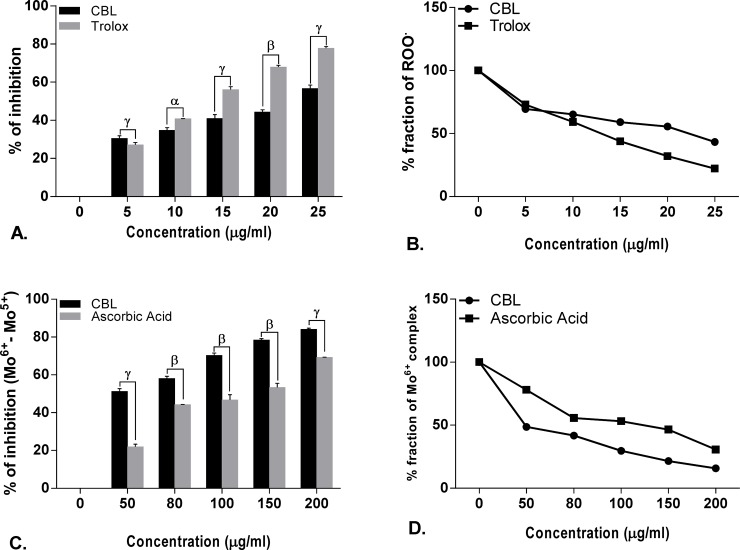
Antioxidant activity of *Croton bonplandianus*. **(A)**&**(B)** concentration dependent Total antioxidant activity and extent of Mo^6+^ reduction; **(C)** % inhibition of lipid peroxidation Vs standard trolox; **(D)** depicts remaining unneutralized lipid peroxides (ROO^●^). Data expressed as mean ± S.D (n = 6). α p<0.05; β p<0.01; γ p<0.001; NS-Non significant when compared with standard.

**Fig 4:**
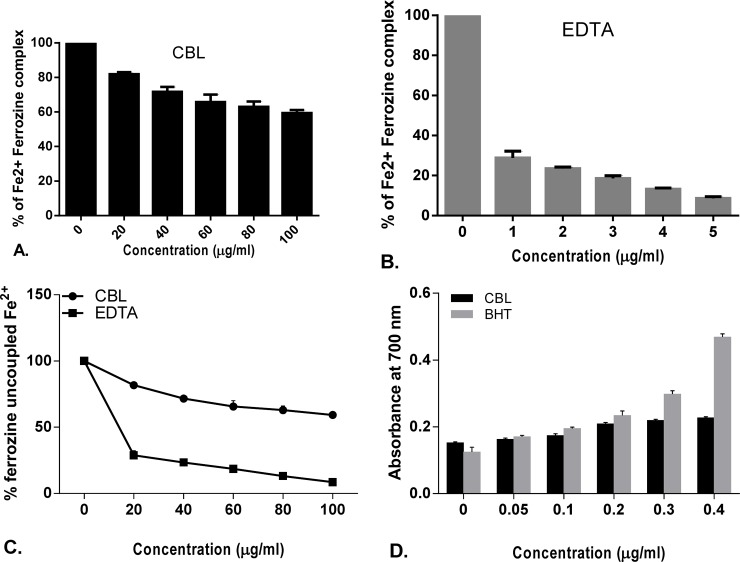
Antioxidant activity of *Croton bonplandianus*. **(A)** Fe^2+^-chelation Vs standard EDTA; **(B)** depicts remaining unneutralized Fe^2+^; **(C)** % inhibition of Superoxide (O_2_^●^ˉ) Vs standard quercetin; **(D)** depicts unneutralized superoxide (O_2_^●^ˉ) radicals. Data expressed as mean ± S.D (n = 6). α p<0.05; β p<0.01; γ p<0.001; NS-Non significant when compared with standard.

**Fig 5:**
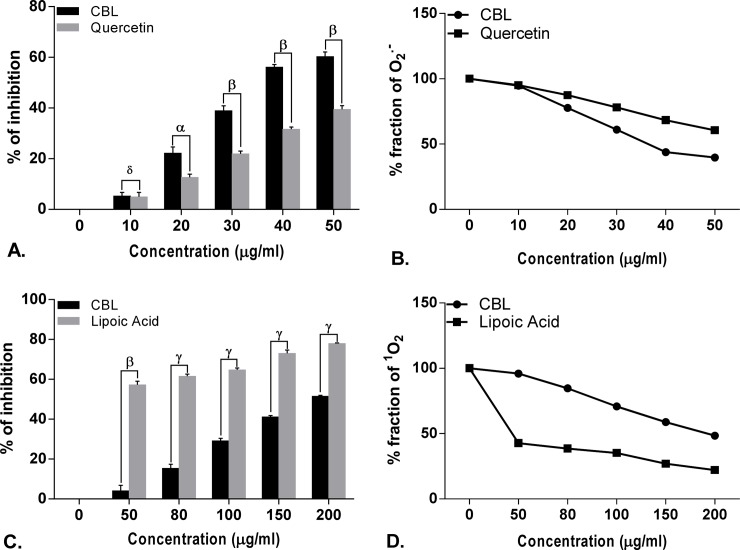
Antioxidant activity of *Croton bonplandianus*. **(A)** % inhibition of Singlet O_2_ (^1^O_2_) Vs standard lipoic acid; **(B)** depicts unneutralized Singlet O_2_ (^1^O_2_); **(C)** % inhibition of Nitric oxide (NO) Vs standard curcumin; **(D)** depicts unneutralized nitric oxide (NO). Data expressed as mean ± S.D (n = 6). α p<0.05; β p<0.01; γ p<0.001; NS-Non significant when compared with standard.

**Fig 6:**
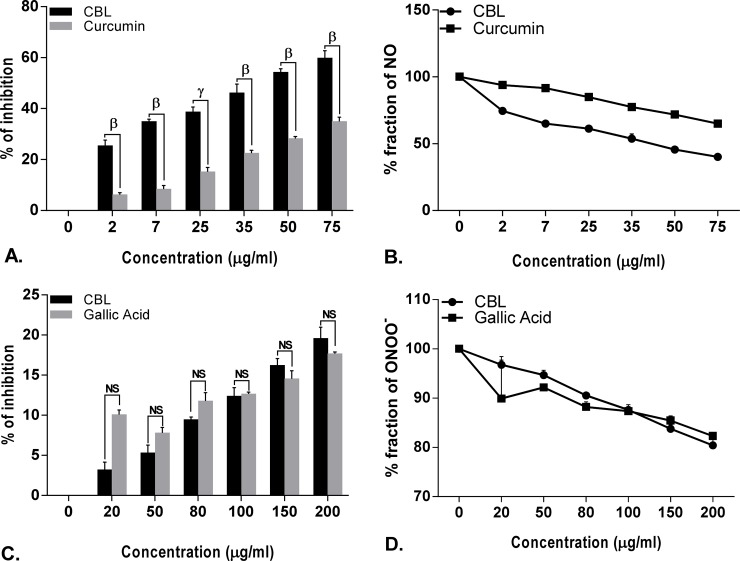
Antioxidant activity of *Croton bonplandianus*. **(A)** % inhibition of Peroxynitrite (OONOˉ) Vs standard gallic acid; **(B)** depicts unneutralized Peroxynitrite (OONOˉ); **(C)** Total reducing power activity.Data expressed as mean ± S.D (n = 6). α p<0.05; β p<0.01; γ p<0.001; NS-Non significant when compared with standard.

Leaf extract of CBL showed lower IC_50_ value than ascorbic acid, mannitol, sodium pyruvate, curcumin, quercetin in DPPH, hydroxyl radical, H_2_O_2_, nitric oxide, superoxide anion, hypochlorous acid scavenging assay and found comparable with peroxinitrate shown by gallic acid and lipid peroxidation shown by trolox. On the other hand, CBL showed higher IC_50_ value than lipoic acid and EDTA in singlet oxygen and iron chelation scavenging assay. In case of total antioxidant activity, CBL showed better scavenging activity than the standard ascorbic acid. The reducing power of CBL was also determined and found that the reducing capacity of CBL extract was increased in a dose dependent manner comparable to the reference compound ascorbic acid (Figs 1–6).

### Differential effects on the individual components of Haber–Weiss reaction (Fenton chemistry)

The Fenton chemistry as a part of the Haber-Weiss reactions is considered central to the intracellular free radical formation cascade. *C*. *bonplandianus* extract was evaluated for their capacity to directly affect the individual components of the Haber–Weiss reaction. *C*. *bonplandianus* demonstrated significantly H_2_O_2_ neutralizing activity (Fig 2A and 2B). The ferric iron (Fe^2+^) chelation capacity of *C*. *bonplandianus* at 200 μg/mL was 59.36 ± 1.81% (Fig 4A and 4B). EDTA, however demonstrated significantly (P<0.001) superior ferric chelation capacity as demonstrated by its low IC_50_ value of 10.23±0.89 μg/ml. In case of direct scavenging of OH●, *C*. *bonplandianus* demonstrated superior activity than that of the standard mannitol (Fig 1C and 1D).

### Inhibition of OH^●^: Bivariate analysis

Dose-dependent bivariate correlation analysis of Fe^2+^ chelation and H_2_O_2_ inhibition were performed vs inhibition of OH● in order to reflect how individually *C*. *bonplandianus* may inhibit the formation of OH●. Fe^2+^ chelation activity by the inhibition of OH^●^ is higher in the presence of *C*. *bonplandianus* (r = 0.8925 and r^2^ = 0.7966). The correlation between the inhibitions of OH^●^ and H_2_O_2_ were much more comparable, with minor trend towards H_2_O_2_ mediated effect (r = 0.9825 and r^2^ = 0.9652) (S1 Fig).

### Determination of phenol and flavonoid content in CBL extract

Total amount of phenoloic content present in the hydromethanolic extract of CBL was found to be 75.06 ± 2.33 mg/ml gallic acid equivalent per 100 mg plant extract and the total flavonoid content of CBL extract was 52.17 ± 4.36 mg/ml quercetin equivalent per 100 mg plant extract.

### Detection of intracellular ROS generation

**Fig 7:**
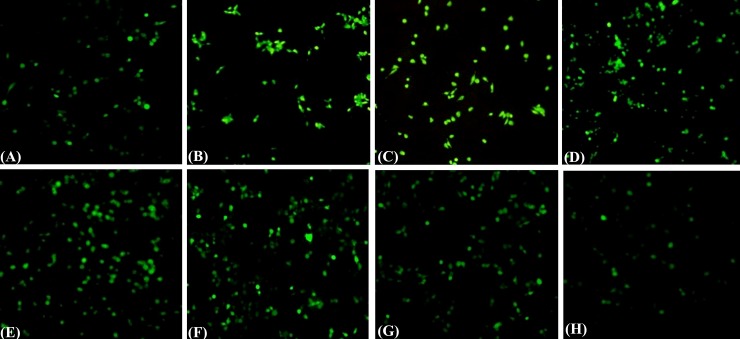
Effects of CBLin depletion of intracellular ROS production generated by CCl_4_ in WRL-68 cells. Production of ROS was measured by cleavage of acetate group of non-fluorescent H_2_DCFDA (2',7'-dichlorodihydrofluorescein diacetate) which convert into DCF(2′,7′ -dichlorofluorescein) highly fluorescent. Cells were exposed to CCl_4_ before treatment with CBL 50, 80, 100, 150 and 200 μg/ml for 24 h. The ROS production displays the intensity of fluorescence through the images of WRL-68 cells treated with different concentration of CBL **(D-H)**, CCl_4_ **(C)**, H_2_O_2_ **(B)** and control **(A)**.

Human hepato cell line WRL-68 was used to examine the effects of CBL under oxidative stress. CCl_4_ increases oxidative stress levels in the liver tissue, and based on that study, it is speculated that CCl_4_may induce the oxidative stress in WRL-68 cells. Therefore, WRL-68 cells were treated with CCl_4_ for 0–24 h, and intracellular oxidative levels were measured using the dichlorofluorescein assay. Fig 7 demonstrated that cells exposed to CCl_4_ exhibited significantly increase in ROS levels. Tremendous decrease in fluorescence was detected at higher doses of CBL (200μg/ml) at 24h post exposure compared to the CCl_4_ (Fig 7). The resulting change in fluorescence intensity gives strength to the hypothesis that CBL affects in the production of intracellular ROS.

### Hepatoprotective activity

#### Acute toxicity study

CBL extract was administrated to the experimental animals up to 2,000 mg/kg body weight. However, at the 2000 mg/kg body weight dose, no sign of mortality and physiological deformation were observed in the experimental animals. Therefore, 50 mg/kg BW, 100 mg/kg BW and 250 mg/kg BW doses were selected as a low, medium and high dose in the *in vivo* hepatoprotective experiments.

#### Body and liver weight

Significant body weight changes were observed in CCl_4_, CBLM and silymarin groups shown in Table 1. Final body weight was decreased only in CCl_4_ groups (12.53 ± 1.39). On the other hand liver weight of CCl_4_ group (5.16 ± 0.15) resulted in the highest relative liver weight (26.37 ± 1.40) among all the groups. Interestingly only high dose group (CBLH) prevented utmost percent of body weight changes. The relative liver weight of all the groups were closes another, except CCl_4_ group.

**Table 1:** Changes of body weight (g) and liver weight (g) in different experimental groups. Data represented as mean ± SD of six observations.

Group	Initial body weight	Final body weight	% body weight change	Liver weight	Relative liver weight
Control	22.82 ± 0.65	25.84 ± 0.18 *	11.69 ± 2.63 ▲	4.70 ± 0.16	18.21 ± 0.68
CCl_4_	22.06 ± 0.29	19.61 ± 0.50 **	12.53 ± 1.39 ▼	5.16 ± 0.15 ^NS^	26.37 ± 1.40
Silymarin	22.71 ± 0.54	24.67 ± 0.85 *	7.92 ± 1.24 ▲	4.52 ± 0.21 ^NS^	18.33 ± 0.57
CBL (50 mg/kg BW)	23.22 ± 0.41	23.87 ± 0.17 *	2.72 ± 1.08 ▲	5.07 ± 0.12 ^NS^	21.24 ± 0.42
CBL (100 mg/kg BW)	22.05 ± 0.52	22.99 ± 0.48 **	4.06 ± 0.32 ▲	4.89 ± 0.25 ^NS^	20.88 ± 0.60
CBL (250 mg/kg BW)	22.73 ± 0.76	24.59 ± 0.41 *	7.57 ± 2.72 ▲	4.63 ± 0.22 ^NS^	18.83 ± 1.12

NS p > 0.05

*p < 0.05

**p < 0.01.

Final body weight was compared with initial body weight of corresponding group and liver weight of treated groups was compared with liver weight of control group. ▲ represents increase and ▼ represents decrease.

#### *In vivo* liver marker enzymes and biochemical parameters

The effect of CCl_4_ and the subsequent administration of silymarin and CBL on the various serum enzymatic and biochemical parameters are shown in Table 2. All the *in vivo* experimental parameters were increased in case of CCl_4_ group and subsequently decreased with silymarin and CBL treatment except protein and albumin.

**Table 2:** Describes the levels of various enzymatic and biochemical parameters in the serum of six (n = 6) treated groups. The data represented as mean ± SD of six observations.

Parameters (units)	Control	CCl_4_	Silymarin	CBL (50 mg/kg BW)	CBL (100 mg/kg BW)	CBL (250 mg/kg BW)
ACP (K.A.)	3.81 ± 0.04	13.13 ± 0.63**	6.02 ± 0.48*^b^	11.21 ± 0.23***^d^	9.88 ± 0.73**^a^	8.21 ± 0.22**^b^
ALP (K.A.)	13.09 ± 0.30	31.16 ± 0.25***	16.23 ± 0.35***^c^	29.30 ± 0.48***^d^	28.24 ± 0.35***^b^	23.07 ± 0.66***^c^
AST (u/ml)	63.34 ± 0.59	142.19 ± .66***	82.66 ± 0.94***^c^	141.70 ± .81***^d^	127.72 ± 0.91***^b^	108.49 ± .11***^c^
ALT (u/ml)	47.94 ± 0.65	137.39 ± .61***	56.53 ± 0.87**^c^	123.18 ±0.39***^c^	105.72 ± 1.11***^c^	77.20 ± 0.32***^c^
GGT (u/l)	3.76 ± 0.11	8.26 ± 0.52**	4.90 ± 0.20*^b^	7.06 ± 0.33**^d^	6.32 ± 0.26**^d^	5.60 ± 0.32*^b^
Glucose (mg/dl)	57.04 ± 1.53	85.18 ± 1.19**	63.85 ± 1.32*^b^	81.72 ± 2.20**^d^	72.15 ± 0.65**^c^	67.41± 0.91**^b^
Protein (g/dl)	5.93 ± 0.06	4.07 ± 0.05***	5.72 ± 0.07^NS^^b^	4.11 ± 0.06***^d^	4.26 ± 0.09***^a^	5.12 ± 0.31*^a^
Albumin (g/dl)	4.54 ± 0.33	2.27 ± 0.20*	3.43 ± 0.17**^a^	2.52 ± 0.13*^d^	2.87 ± 0.10*^d^	3.03 ± 0.16*^d^
Globulin (g/dl)	2.19 ± 0.02	0.84 ± 0.03***	1.99 ± 0.03*^c^	0.99 ± 0.03***^b^	1.16 ± 0.03***^c^	1.72 ± 0.04**^c^
Bilirubin (mg/dl)	0.81 ± 0.04	2.04 ± 0.07**	1.05 ± 0.05**^b^	1.88 ± 0.09**^d^	1.71 ± 0.04**^b^	1.33 ± 0.04**^b^
Urea (mg/dl)	35.43 ± 3.94	128.76 ± 6.38**	58.16 ± 4.76*^b^	116.46 ± 4.29***^d^	100.51 ± .68**^b^	78.24 ± 3.04**^b^
Urea N_2_ (mg/dl)	12.69 ± 0.96	72.55 ± 1.91***	30.65 ± 0.91***^b^	68.05 ± 1.38***^a^	53.70 ± 1.45***^b^	40.61 ± 1.24**^b^
LDH (u/l)	240.73 ± 2.89	571.83 ± .41***	296.01 ± 2.15**^c^	548.55 ± 4.45***^c^	502.66 ± 6.04***^b^	340.50 ± .67***^c^
Cholesterol (mg/dl)	68.66 ± 1.51	132.17 ± .38***	90.37 ± 2.05**^c^	113.38 ± 3.55***^b^	101.35 ± 2.19**^b^	93.22 ± 1.67**^b^

^NS^P = non significant (p> 0.05)

*P < 0.05

**P < 0.01 and

***P < 0.001vs control group; whereas

^d^P = non significant (p> 0.05)

^a^P < 0.05

^b^P < 0.01 and

^c^P < 0.001 vs CCl_4_ group.

#### Estimation of hepatic antioxidative enzymes: Catalase, peroxidase, superoxide dismutase and reduced glutathione

**Fig 8:**
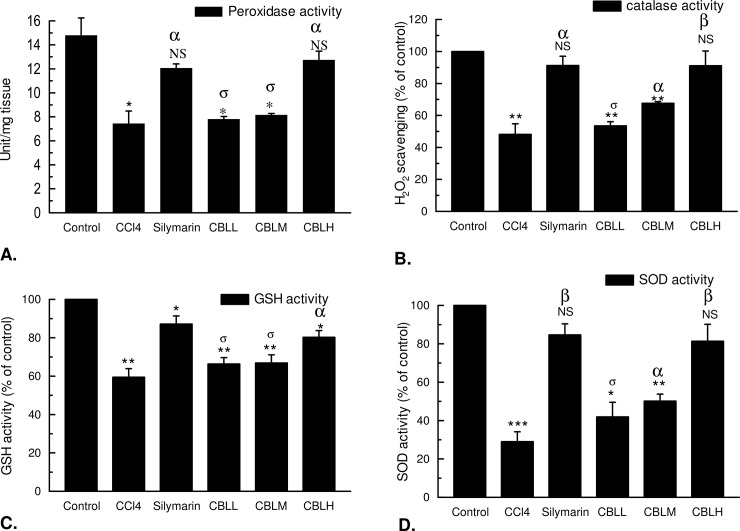
**The effect of *Croton bonplandianus* on the (A)** Peroxidase; **(B)** Catalase; **(C)** Reduced glutathione (GSH); **(D)** Superoxide dismutase (SOD) activities in CCl_4_ intoxicated liver samples. Comparisons were made with control for statistical inference (‘t’ test for paired comparison) to interpret significant difference. Data expressed as mean ± S.D (n = 6). ^α^ p<0.05; ^β^ p<0.01; ^γ^ p<0.001; ^NS^-Non significant.

Significant inhibition of enzymatic catalase and SOD (superoxide dismutase) and non-enzymatic reduced glutathione by CBL extract occurred in CCl_4_ intoxicated mice when compared with the control (Fig 8). CBL treatment enabled significant increase in the enzyme activity of catalase and reduced glutathione when compared with CCl_4_ toxicated groups. On the other hand silymarin treatment significantly increase the percent of inhibition compared with the CCl_4_ treated mice. On the other hand the activity of peroxidase enzyme in hepatic tissue is significantly lowered as a result of CCl_4_ treatment (Fig 8). The peroxidase activity in the control group was 14.79 unit/mg tissues which were lowered 7.43 unit/mg tissues due to CCl_4_ administration. The lowered peroxidase activity was significantly elevated by CBLH (12.74 unit/mg tissue) when compared with the standard silymarin treated group (12.07 unit/mg tissue).

#### *In vitro* liver marker enzymes and biochemical parameters

The hepatoprotective potential of CBL extract was reflected through *in vitro* liver marker enzymes and biochemical parameters. The results were compared with the standard drug silymarin as shown in Table 3. The CCl_4_ group showed the higher toxicity than the other groups.

**Table 3:** Changes in the levels of various enzymatic and biochemical parameters of the culture supernatants of the experimental groups. Data represented as mean ± SD of six observations.

Parameters (units)	Control	CCl_4_	Silymarin	CBL (50 mg/kg BW)	CBL (100 mg/kg BW)	CBL (250 mg/kg BW)
ACP (K.A.)	0.80 ± 0.04	1.95 ± 0.04***	1.21 ± 0.03**^b^	1.72 ± 0.03***^a^	1.58 ± 0.04**^a^	1.36 ± 0.05**^b^
ALP (K.A.)	3.38 ± 0.05	8.06 ± 0.07***	4.92 ± 0.23**^c^	6.83 ± 0.14***^b^	6.22 ± 0.12**^b^	5.45 ± 0.23**^b^
AST (u/ml)	15.54 ± 0.66	54.63 ± 0.75***	20.32 ± 0.94**^c^	43.98 ± 0.52***^d^	40.30 ± 1.11***^b^	28.52 ± 0.85**^b^
ALT (u/ml)	7.71 ± 0.34	34.57 ± 1.64**	13.95 ± 0.29***^b^	29.29 ± 0.77***^d^	26.28 ± 0.80***^a^	18.42 ± 1.00**^c^
GGT (u/l)	0.64 ± 0.03	1.16 ± 0.03***	0.79 ± 0.04*^b^	1.08 ± 0.03***^d^	1.03 ± 0.01***^a^	0.913 ± 0.02***^b^
Bilirubin (mg/dl)	0.21 ± 0.02	0.67 ± 0.02***	0.316 ± 0.02**^c^	0.64 ± 0.03***^d^	0.54 ± 0.02***^c^	0.41 ± 0.02**^b^
Protein (g/dl)	7.49 ± 0.04	5.69 ± 0.27**	7.05 ± 0.06**^a^	5.99 ± 0.03***^d^	6.63 ± 0.27*^a^	6.91 ± 0.16*^b^
LDH (u/l)	42.59 ± 1.10	211.01 ± 2.59***	121.18 ± 1.36***^c^	188.94 ± 2.78***^a^	167.48 ± 3.04***^b^	135.20 ± 1.49***^c^

^NS^P = non significant (p> 0.05)

*P < 0.05

**P < 0.01 and

***P < 0.001vs control group; whereas

^d^P = non significant (p> 0.05)

^a^P < 0.05

^b^P < 0.01 and

^c^P < 0.001 vs CCl_4_ group.

#### Lipid peroxidation (MDA level)

**Fig 9:**
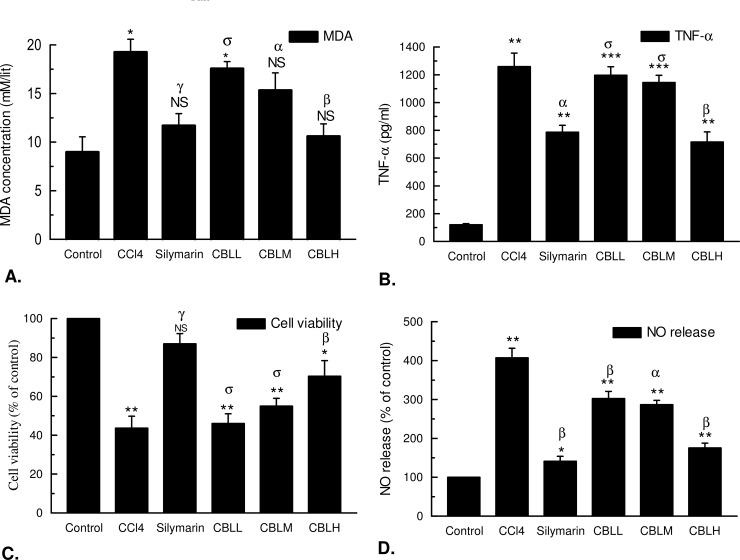
**The effect of *Croton bonplandianus* on (A)** MDA level; **(B)** TNF-α level; **(C)** Cell viability; **(D)** NO release activities in CCl_4_ intoxicated liver samples. Comparisons were made with control for statistical inference (‘t’ test for paired comparison) to interpret significant difference. Data expressed as mean ± S.D (n = 6). ^α^ p<0.05; ^β^ p<0.01; ^γ^ p<0.001; ^NS^-Non significant.

Lipid peroxidation or MDA level in the treated groups are illustrated in (Fig 9A). The MDA content was elevated from 8.99 μM/litre in control to 19.27 μM/litre in CCl_4_ group. Significant results found when the elevated MDA level was lowered to10.60 μM/litre after CBLH administration.

#### MTT cytotoxic effect

Viability of cells were decreased in the CCl_4_ group significantly (p<0.01) compared to the control group (Fig 9C). The viability of the cells in CCl_4_ group was only (43.67 ± 6.11), where in the standard silymarin group the percent of viability of cells was (87.00 ± 5.29) which was very close to the control group. On the other hand, in the experimental groups, the cell viability was increased gradually. Percentage of cell viability in CBLH group (70.33 ± 8.02) was very close to the standard group.

#### Measurement of the release of TNF- α

Measurement of TNF- **α** release are demonstrated in the Fig 9B. The level of TNF- **α** in control group was 120.32 ± 8.04 pg/ml, which was increased 1259.20 ± 96.96 pg/ml due to CCl_4_ toxicity. However the TNF-α level decreased better by CBLH group (716.66 ± 73.06 pg/ml) when compared with the standard silymarin group (786.22 ± 49.70 pg/ml).

#### Inhibition of nitric oxide (NO)

CCl_4_ toxicity resulted increases in NO release when compared to the control (Fig 9D). However, significant (p < 0.001) lowering of NO level was observed in the treated groups. The NO level in silymarin and CBLH groups were 141.33 ± 12.70 and 175.33 ± 12.50% respectively, when NO release of control was considered as 100%.

### Histopathological examination

**Fig 10:**
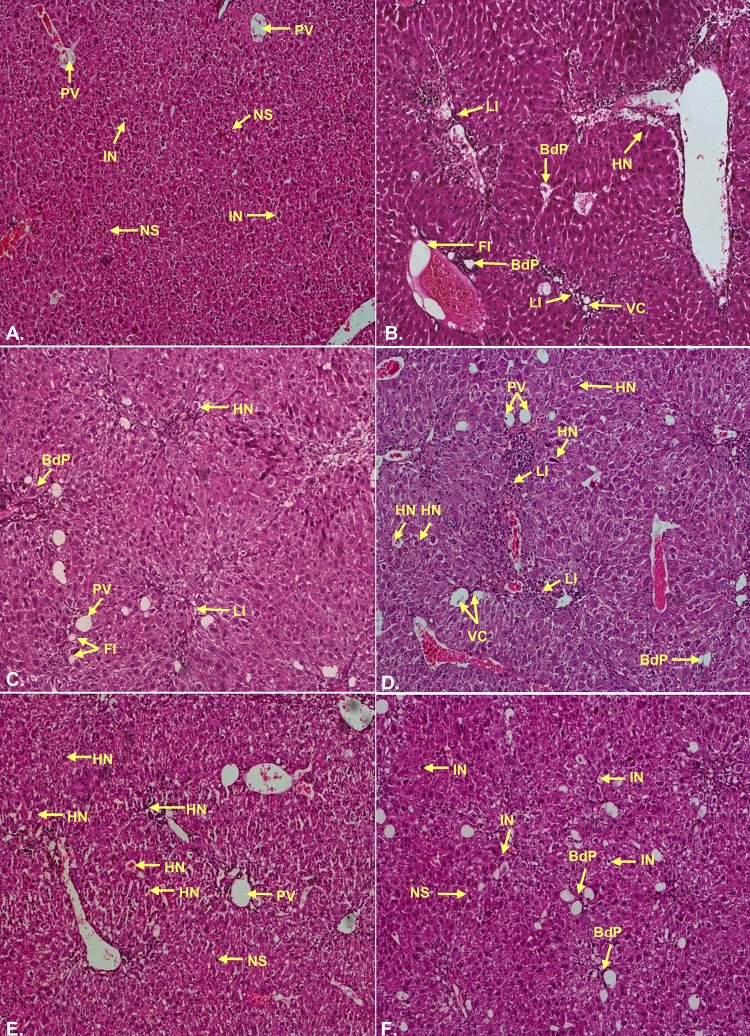
Photomicrographs (100×) of the histopathological examinations of the liver samples of different groups. **Even though the extract treated groups possessed injury marks however, the extent of signs of injury were much lower in the extract treated groups compared to CCl**_**4**_ **group.(A)** Control group liver demonstrated normal liver architecture with normal sinusoids (NS), hepatocytes with intact nucleus (IN), un-inflamed portal vein (PV); **(B)** CCl_4_ group liver demonstrated significant loss of hepatocellular architecture with extensive fatty infiltration (FI) leading to steatosis, bile duct proliferation (BdP), vascular congestion (VC) and haemorrhagic necrosis (HN) around portal vein. Loss of hepatic nodular structure and disorganized hepatocytes marked the CCl_4_ induced liver damage; **(C)** Silymarin group demonstrated hepatoprotective activity by substantial amendment of proliferated bile duct (Bd) with normal sinusoids (NS) and intact portal veins (PV); **(D)** CBLL group was marked by less leukocyte infiltrations (LI), sinusoidal dilations (SD) and bile duct proliferation (BdP); **(E)** CBLM group reflected comparatively less haemorrhagic necrosis (HN) and fatty infiltrations (FI); **(F)** CBLH group demonstrated lowering of most of the injury signs however, leukocyte infiltrations (LI) could be identified in the liver samples.

**Fig 11:**
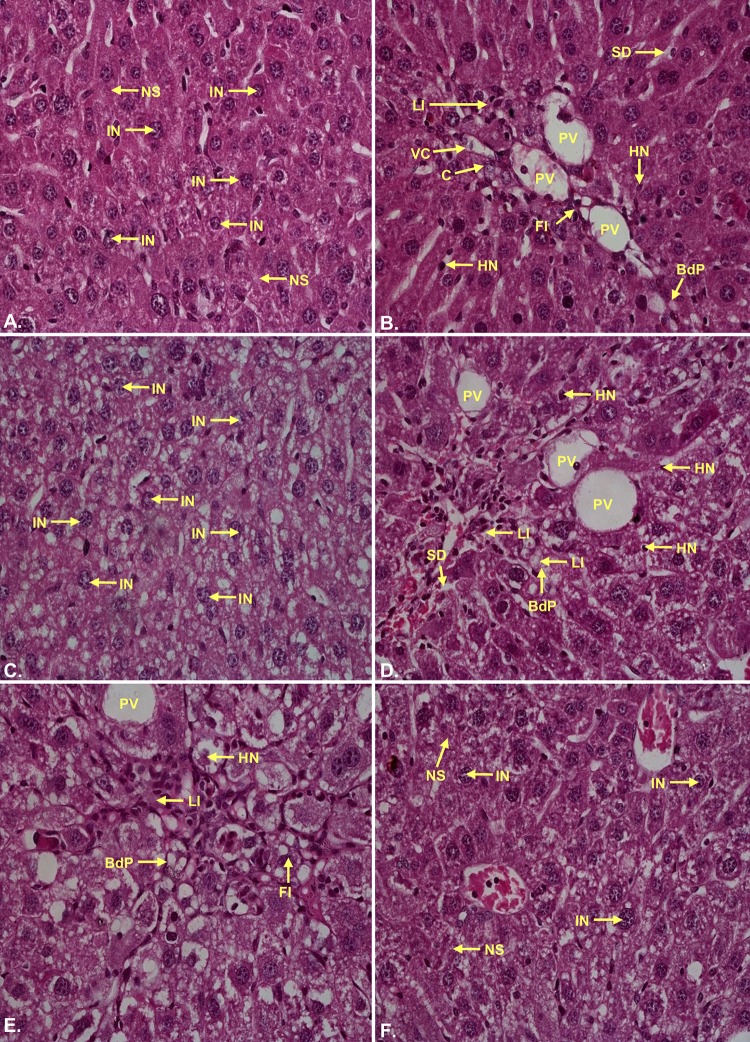
Photomicrographs (400×) of the histopathological examinations of the liver samples of different groups. **(A)** Control group liver sampled possessed well packed hepatocytes with intact nucleus (IN) and normal sinusoids (NS); **(B)** CCl_4_ group liver possessed extensive fatty infiltrations (FI), Necrotic hepatocytes (N), prominent signs of inflammation with leukocyte infiltrations (LI), prominent calcification (C) around the congested vesicles (VC) with bile duct proliferations (BdP); **(C)** Silymarin group liver samples were characterized with normal sinusoids (NS) and intact nucleus (IN) containing healthy hepatocytes; **(D)** CBLL group demonstrated lower fatty infiltrations (FI), sinusoidal dilations (SD) and leukocyte infiltrations (LI); **(E)** CBLM group resulted in renewal of normal hepatic architecture with several hepatocytes with intact nucleus (IN) and lowered sinusoidal dilations (SD); **(F)** CBLH group showed near to normal hepatic architecture with predominantly intact nucleus (IN) containing normal hepatocytes and undiluted normal sinusoids (NS).

There are several hiotological parameters showed the injury level of experimental groups as enlisted in S2 Table. The haematoxilin-eosin staining of hepatocytes displayed clearly the well maintained hepatocellular integrity, healthy cellular architecture, and clear cytoplasm with prominent nucleus in the control group. But in the CCl_4_ group, several damages have been observed. Hepatocytes of the CCl_4_ groups showed hepatocellular necrosis, bile duct proliferation, sinusoidal dialation, inflammation (leukocyte infiltration), vascular congestion, loss of structure of hepatic nodules, fatty infiltration, vascular degeneration and calcification. Most strikingly fibrosis, the thickening and scoring of connective tissue, as a result of injury was notified in the CCl_4_ group (Figs 10 and 11; S2 Fig). The injury level found in the CCl_4_ group was down regulated by the administration of standard drug silymarin. Interestingly, in the present study it was observed that high dose of plant extract (CBLH) down regulates the injury better or similar compared to the standard silymarin.

### GC-MS analysis

**Fig 12:**
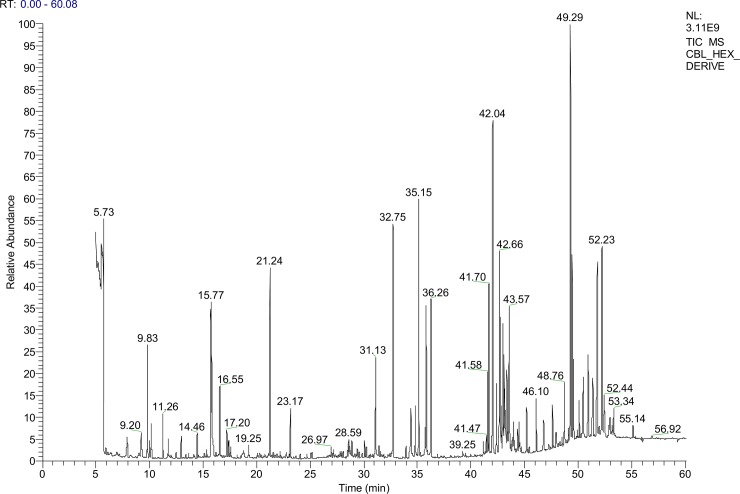
Gas Chromatography Mass Spectrometry (GC-MS) analysis of *Croton bonplandianus*.

GC-MS analysis showed the chemical fingerprint of the CBL extract (Fig 12 and S3 Table). The GC-MS data reported that the presences of several bioactive compounds, of which many of them are documented, possess distinct and definitive pharmacological activities.

### Molecular docking

**Fig 13:**
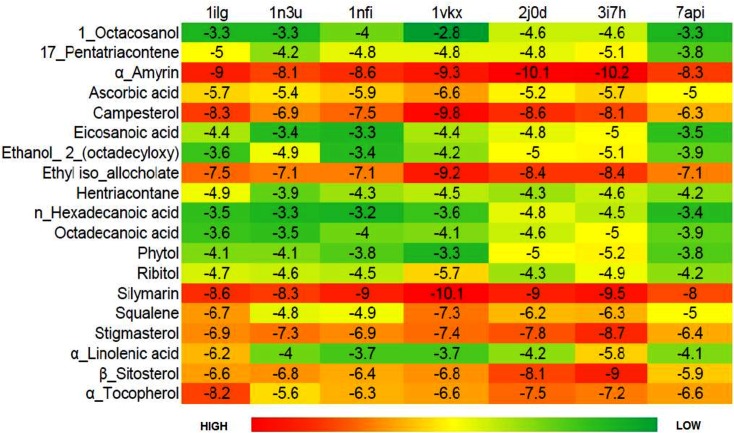
Heatmap based on binding energy among proteins and phytochemicals. The phytochemicals which served as ligands for molecular docking experiment are along the Y axis and the proteins are placed on the X-axis.

**Fig 14:**
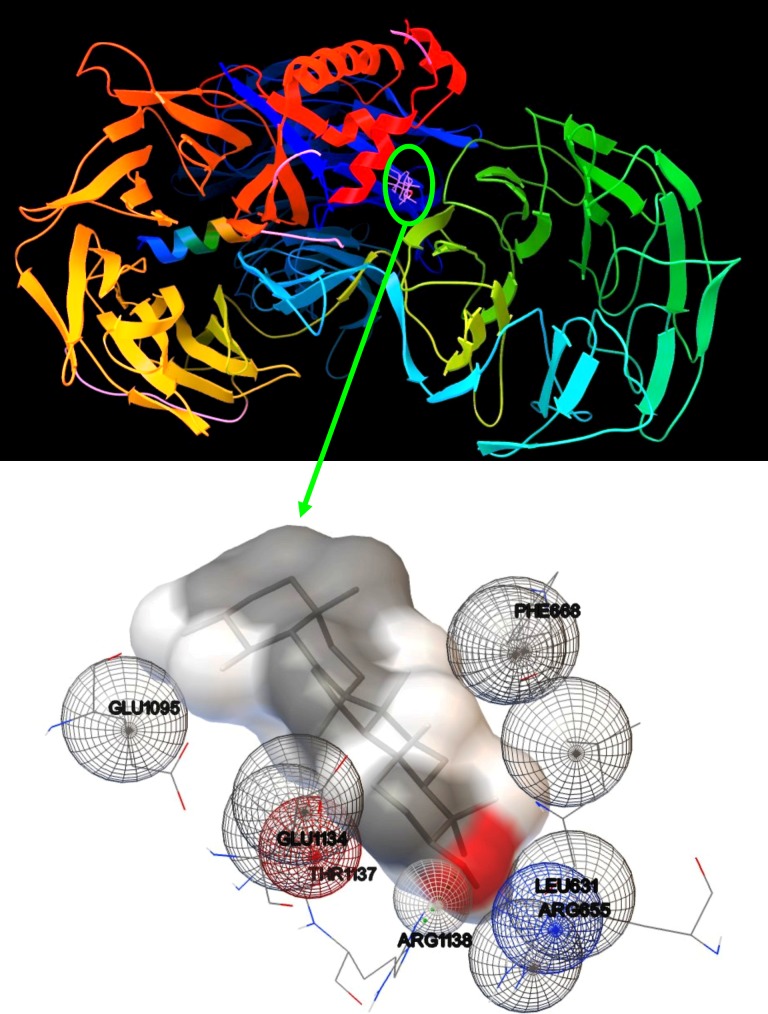
Molecular docking (secondary structure view) between Hepatitis BX protein and α-amyrin.

The bioactive compounds of *Croton bonplandianus* was checked for possible interactions with several proteins playing the essential role in different metabolic pathways of humans and other major vertebrates. The proteins were chosen those have relationship with the health of the liver. These proteins acted as receptors required for molecular docking experiments. The ligands required to conduct the experiment are the compounds identified my GC-MS analysis of the plant extract. Upon a series of receptor-ligand interaction study, it was identified that each of the ligands has different binding affinity with the selected proteins. It is seen that α-amyrin has the highest interaction with all the receptors on an average followed by Campesterol and Ethyl iso-allocholate (Fig 13). On the other hand 1- Octacosanol has the least binding affinity with the receptors. For the comparative analysis a standard was used. Silymarin a proven drug against hepato toxicity was used as a standard in this regard. One of the phytochemical α-amyrin had a binding affinity better than silymarin with all the receptors on average. The highest binding affinity was found between α-amyrin and a protein with PDB ID 3i7h which is the crystal structure of DDB1 in complex with H-Box Motif of HBX (Fig 14). NFκβ protein and Campesterol also has good binding affinity and as seen in the molecular surface view of the protein moiety the ligand binds nicely inside a cavity in the protein surface (S3 Fig).

## Discussion

The demand of late medicine or health supplement of natural origin has increased many folds because of their potential to prevent and reduce the risk of several oxidative damage with minimal side effects [32]. Antioxidant property covers a broad spectrum of chemical phenomenon and definite antioxidant activity should not be concluded based on a single experimental model. Therefore, in practice several *in vitro* antioxidants or free radical scavenging activities were carried out with our sample of interest. In the present antioxidant profiling, *C*. *bonplandianus* leaf extract showed potential free radical scavenging activities. The molecule DPPH is a free radical that can accept an electron or hydrogen radical to become stable and reacts with reducing agent to form new bond, changing the color of the solution. The colored DPPH solution mixed with natural antioxidants. DPPH gives rise to the reduced form with the loss of violet color by the effect of natural antioxidants. Thus, DPPH scavenging activity by CBL extract proves the presence of significant antioxidant properties. Human beings are exposed to H_2_O_2_ indirectly via environment. This, H_2_O_2_ may enter into the human body by normal physiological function. Inhibition of H_2_O_2_ indirectly from environment is rapidly decomposed into oxygen and water and this may produce hydroxyl radicals (OH^.^) that can cause lipid peroxidation and DNA damage in the body. Therefore, the ability of CBL extract to scavenge H_2_O_2_ proves beneficial for our health. Nitric oxide plays an important role as pro-inflammatory mediators. Nitric oxide (NO^.^) is synthesized from the amino acid L-arginine by the activation of nitric oxide synthase (NOS). During chronic inflammation iNOS (Calcium independent isoform of NOS) is activated by LPS (Lipopolysaccharide) and produces huge amount of nitric oxide. The active NO^.^ translocate NF-κβ and leads to the formation of cancer. In mitochondria excess amount of nitric oxide reacts with superoxide radical to produce reactive peroxynitrite radical which further cause oxidative stress related disorder. In the present study, it is demonstrated that nitric oxide is down regulated by CBL extract when compared to standard silymarin. Thus, *C*. *bonplandianus* might inhibit the inflammation related disorders. On the other hand peroxynitrate (OONO^.^), a reactive nitogen species containing free radical, is a cytotoxic agent with strong oxidizing properties. The oxidizing properties of peroxynitrate (OONO^.^) towards various cellular constituents including amino acids, lipid, nucleotide can cause cell death, lipid peroxidation and alleviating chances of carcinogenesis. Therefore, peroxynitrate scavenging activity by CBL extract is beneficial for health. Hydroxyl radical generated from hydrogen peroxide by Fenton reaction is one of the potent reactive oxygen species in the biological system that react with phospholipids containing polyunsaturated fatty acid moieties of cell membrane and cause damage of cell [33]. Hypochlorous acid produced from the site of chronic inflammation resulting from the oxidation of Cl^-^ ion by the neutrophil enzyme, myelo-peroxidase. Hypochlorous acid degrades heme prosthetic group and inactivates the antioxidantenzyme catalase. Leaf extract of *C*. *bonplandianus* also prove that it has the potentiality to scavenge proxynitrate, hydroxyl radical, superoxide, singlet oxygen and other free radicals that cause the harmful effect in our biological system. Thus, CBL extract might prove to be a key component in prevention of various diseases related to oxidative stress and free radical generation. Keeping in mind the crucial role played by oxidative stress in liver disease, medicinal plant derived antioxidant can clearly be considered as a good therapeutic strategy. In the present study it is tried to establish how antioxidants are linked with hepatic damage or disorder. For this above mentioned study CCl_4_ (Haloalkane) waschosen to induce hepatic damage in murine model amelioration by the leaf extract of *C*. *bonplandianus*was investigated through antioxidant and anti-inflammatory activities. The toxicity profile of CCl_4_ is well established worldwide [34–36]. Extensive usage of CCl4 in industrial sectors has a rich history of environmental toxicity and occupational hazards. This had lead to awareness in the industrial and domestic use of CCl_4_ from the early 70’s, leading to the production and import of CCl_4_ [37]. Multiple sources for generation of reactive oxygen species (ROS) have been identified; among them CCl_4_ was used in the present study as a source for intracellular production of ROS. Hydrogen peroxide (H_2_O_2_) is a stable free radical having important role in signalling pathways [38]. Increased levels of ROS productions are associated with oxidative stress in cell. H_2_DCFDA was used to detect the production of intracellular ROS generation. H_2_DCFDA detects hydrogen peroxide by exhibiting fluorescence on WRL-68 cell line exposed to H_2_O_2,_ suggesting a H_2_O_2_ induced oxidative stress. Generally, CCl_4_ contribute to increase in ROS level. However, a substantial reduction in fluorescence intensity was seen with the increase in concentration of CBL from 50–200μg/ml. This suggests that under the influence of CBL, CCl_4_ induced ROS was diminished proportionately. It can be inferred that CBL plays an important role in reducing the impact of CCl_4_ on normal intracellular function.

**Fig 15:**
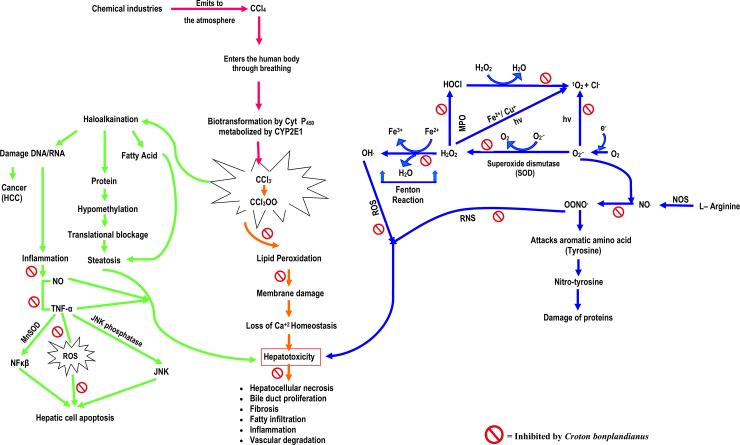
Schematic representation showing the free radical generation followed by the chain of by-product (ROS/RNS) formed due to oxidative stress and how they affect biological systems by cellular stress and CCl_4_ induced hepatotoxicity. The pathway demonstrates the mechanism of CCl_4_ induced hepatotoxicity which is predominantly mediated by oxidative stress and inflammatory injury due to the formation of reactive metabolic intermediates and the free radical formation cascade during xenobiotic induced hepatotoxicity causing oxidative and nitrosative stress. Cyt P450 = cytochrome P450, CCl_3_^●^  = trichloromethyle radical, CCl_3_OO^●^  = trichloromethylperoxy radical, TNF-α  = tumor necrosis factor-α,; HOCl: Hypochlorous acid; H_2_O_2_: Hydrogen peroxide; ^1^O_2_: Singlet oxygen; O_2_^.-^: Superoxide; OH^.^: Hydroxyl radical; 8-OHdG: 8-hydroxy2-deoxy guanosine; OONO^-^: Peroxynitrate; NO^.^: Nitric oxide; Fe^2+^: Iron ion; Cu^+^: Copper ion; Cl^-^: Chlorine ion; ROS: Reactive oxygen species; RNS: Reactive nitrogen species; NOS: Nitric oxide synthase; MPO: Myeloperoxidase; MDA: Malondialdehyde.

Carbon tetrachloride (CCl_4_) induced hepatoxicity is caused to some extent by the partial pressure of reactive oxygen in tissues. Low partial pressure of oxygen results in the formation of CCl_3_^*^ and CHCl_2_^*^ radicals [39,40]. Metabolism of lipid is hampered by CCl_4_ and cause steatosis or fatty liver. On the other hand, high partial pressure of oxygen shifts CCl_4_ metabolisms towards the formation of CCl_3-_OO^*^radical with consequent lipid peroxidation and lead the cells from steatosis into apoptosis [39,41]. In CCl_4_ induced liver injury model, oxidative stress can provoke and promote lipid peroxidation that damage the hepatocellular membrane [39]. This hepatocellular damage is followed by the release of pro-inflammatory chemokines and cytokines (Fig 15) [42].

Now a day’s CCl_4_ is required for the synthesis of chlorofluorocarbons (CFCs) that are used as heat transfer agents in refrigerating equipments and as aerosol propellants. In United States, CCl_4_ has been widely used for industrial and domestic cleaning and sterilisation. There are many cells like kupffer cells, hepatic stellate cells and endothelial cells those are more sensitive to oxidative stress related molecules. TNF-α can be produced in kupffer cells by oxidative stress, which might increase inflammation and apoptosis (Fig 15). In studied animal model significant (P<0.001) loss of body weight and relative liver weight have occurred after CCl_4_ toxicity. After the treatment with CBL extract, the changes in the final body weight were much less compared to control and sylimarin group. This result indicates CBL extract has the potentiality in restricting drastic body weight changes through anti-hyperlipidimic activity. Biomarker of hepatotoxicity represents the altered levels of hepatobillary enzymes transaminase and phosphatase. Whereas normalization of these enzymatic parameter represent the improvement of normal liver function [43–45]. Significant elevation of ACP, ALP, AST, ALT, GGT, LDH, glucose, urea, globulin, bilirubin and cholesterol levels and subsequent liver injury are caused due to CCl_4_ toxicity. All the parameters were subsequently normalized to certain extent due to the sylimarin extract and CBL extract administration. Cultured liver cells can serve as a model for evaluation of *in vitro* hepatotoxicity because of its similarity between intact hepatic systems [46]. The *in vitro* enzymatic result also supports the hepatoprotective potentialities of the plant extract. CCl_4_ is biotransformed by CYP2E1. CYP2E1 is a member of cytochrome P_450_ mixed function oxidase system. CYP2E1 is involved in the metabolism of xenobiotics in the body to produce CCl_3_^.^ and CCl_3_OO^.^, and as a result of that tremendous hepatocellular necrosis is casued. Zonal haemmorrhagic necrosis around the portal veins in the CCl_4_ group demonstrated the hepatocellular injury. Hepatic injury was also supported by MTT cell viability assay which showed loss of cell viability due to CCl_4_ toxicity. However significant improvement was observed after the treatment with CBL extract. Generation of oxidative stress due to CCl_4,_ deactivates the cellular anti-oxidative enzymes [47]. Peroxidase, catalase and superoxide dismutase are the major anti-oxidative enzymes responsible for the neutralization of free radicals. Hydrogen peroxide and lipid peroxides converts into non reactive species by the action of peroxidase enzyme. On the other hand catalase prevents the formation of highly reactive OH^.^ by scavenging H_2_O_2,_ the key molecule of fenton reaction. SOD (super oxide dismutase) alternatively catalyzes the dismutation of superoxide radicals into ordinary molecular oxygen or hydrogen peroxide. Glutathione is a major anti-oxidant enzyme that can also serve as a redox or cell signaling regulator and guard the cells against oxidative injury by reducing H_2_O_2_ and scavenging reactive oxygen and nitrogen radicals. CCl_4_ derived trichloromethyl peroxy radicals (CCl_3_OO^.^) accept the proton from polyunsaturated fatty acid in the biological membrane and cause lipid peroxidation and inhibition of oxidative enzymes. By the inhibition of anti-oxidative enzymes, there is accumulation of O_2_^**.-**^and H_2_O_2_ which is cascade phenomenon of free radical formation and cause hepatic damage [47]. Due to high levels of polyunsaturated fatty acid and transition metals, lipid membranes are vulnerable to oxidative stress and nitrosative stress and this transition metals such as iron are capable of damaging nuclear protein, DNA, inhibit enzymes and degrade lipid membrane through oxidative Haber-Weiss reaction [48–50]. CCl_4_ toxicity markedly increases oxidative stress, lowering liver anti-oxidative enzymes. In this study it is established that the diminished catalase, peroxidase and superoxide dismutase levels and elevated MDA levels were subsequently normalize by CBL administration.

Liver disease/failure is accompanied by the up and down inflammatory conditions. TNF-α and NO plays a major role as pro-inflammatory mediators during oxidative stress related liver injury which leads towards the apoptotic cell death and fibrosis [46,51]. Kupffer cells secret a vast array of cytokines (TNF-α, IL-1, IL-6, IL-8), chemokines (KC/GRO, IP-109, MIP-2, MCP-1) and pro-inflammatory mediators like NO which initiates hepatic inflammation and toxicity under such xenobiotic induced hepatotoxic condition. Reactions of the hepatotoxicity and fibrinogenesis are initiated by the action of TNF-α and the overproduction of NO resulting in endotoxin shock and inflammatory hepatic injury [51–53]. Excess amount of NO couples with O_2_^**.-**^ to generate highly reactive ONOO^-^ in the liver mitochondria. In the present study CCl_4_ toxicity resulted significant (P<0.001) increase in TNF-α and NO levels, those were significantly lowered by the administration of CBL extract. These results proved that leaf extract of *C*. *bonplandianus* exhibited potent anti-inflammatory activities through the suppression of pro-inflammatory mediators of chronic hepatotoxicity.

A key aspect of liver injury is the role of GSH (reduced glutathione) in response to exogenously and endogenously imposed stress by redox reaction. This stress activates various signal transduction and transcriptional pathways. TNF-α is a crucial cytokine which mediates liver injury (Fig 15). The binding of soluble TNF-α to TNFR1 on the plasma membrane of hepatocytes trigger the exposure of cytoplasmic death domain of TNFR1 to form complex 1, which activates NF-κβ, JNK and P^53^ cascade to propagate inflammation and survival signaling (Fig 15). GSH is depleted due to CCl_4_ toxicity after the susceptibility of hepatocytes by TNF-α [54, 55]. The altered GSH level due to CCl_4_ toxicity was subsequently controlled by the administration of CBL extract. The hepatoprotective potentialities of leaf extract of *C*. *bonplandianus* were further established by detailed histopathological study. The results clearly demonstrated that due to CCl_4_ toxicity hepatic architectures were deformed and subsequently attenuated by CBL extract. CCl_4_ toxicity initiates tremendous hepatocellular damages like hepatocellular necrosis, bile duct proliferation, leukocytes infiltration (inflammation), vascular congestion, loss of structure of hepatic nodules, hepatocellular fibrosis, fatty acid infiltration, vascular degeneration and calcification. All these were normalized by the action of CBL extract. Phytochemical constituents were further identified using FTIR and GC-MS analyses for the potent hepatoprotective potentialities.

The constituents of the plant extract were detected by GC-MS and FTIR. In biochemical terms these phytochemicals are the constituents of CBL extract, responsible for all the exciting results obtained so far in this study. This result suggests the presence of acitve biochemicals in the plant extract. To further understand these bioactive molecules on a molecular level *in silico* methods like Molecular Docking experiments were carried out. The bioactive chemicals treated as ligand showed overall good binding affinity with the proteins taken as receptors for the molecular docking experiments (Fig 13). Among the receptors, Hepatitis BX (PDB ID 3I7H) protein showed best binding affinity with the phytochemicals (Fig 13).Hepatitis BX may act as the precursor for Hepatocellular carcinoma (HCC). Hepatitis BX promotes the expression of insulin-like growth factor (IGF) in HCC [56]. Thus blocking this protein with this phytochemical can reduce the chances of development of HCC in case of liver diseases. The protein with second highest binding affinity is of Human Cytochrome p450 3A4. Cytochrome p450 3A4 is the major isozyme in the human liver. Proteins like preganane X and NF-κB also showed good interactions with these phytochemicals. Preganane X whose primary function is to sense the presence of alien toxic substances and in response up-regulate the expression of proteins involved in the detoxification and clearance of such substances from the body. NF-κB controls cytokine production and cell survival, but in certain cases its regulation is related to cancer, inflammation and autoimmune diseases. Phytochemicals from the CBL extract act as suitable ligand for all these receptors. So whether it is because of the individual bioactive phytochemical or the result of synergestic effects of all the biochemicals, the plant can be considered to have medicinal benefits against hepatotoxicity. Silymarin, a potent antioxidant and hepatoprotective agent, is used as standard for molecular docking. It is seen that a compound named α-Amyrin is present in CBL extract as detected by GC-MS procedure; α-Amyrin has best binding affinity with all our receptors. Even it showed better results than silymarin. So, even for those who does not agree with synergistic effect of compounds in herbal medicine, α-Amyrin has a point to prove as it shows better molecular binding affinity than a already established drug.

## Conclusion

The current study reports the first ever detailed anti-oxidant and hepatoprotective evaluation of ethnomedicinal plant *Croton bonplandianus*. Oxidative stress can arise from overproduction of ROS by metabolic reaction. Overproduction of ROS uses oxygen and shifts the balance between oxidant/anti-oxidant status. In recent years free radicals such as NO, ONOO^-^, H_2_O_2_, O_2_^.—^and OH^.^ are the prime that mediate oxidative stress emerge as the corner-stone or precursor of several harmful diseases. These free radicals take part in immune reaction during chronic pro-inflammatory response, causing the tissue damage. On the other hand overproduction of these reactive oxygen and nitrogen species are involved in liver damage. CCl_4_ induced liver toxicity is a multidimensional phenomenon predominantly governed by free radicals and inflammatory related responses. Inflammation and oxidative stress are closely linked with each other and one of them may appear before or after the other. But when one of them appears the other, one is most likely to appear and take part in the pathogenesis of many chronic diseases including liver damage. In the present study it is evident that CBL extract aided in the recovery of oxidative stress, liver enzymatic levels, normalize hepatic anti-oxidative liver enzymatic levels, inhibited lipid peroxidation and cell death. On the basis of these facts, antioxidant therapy by CBL extract alone or in combination with other pharmacological strategies appear as the most reasonable treatment of CCl_4_ induced hepatic damage and provides a better means of treating various hepatic complications in future.

## Supporting information

S1 Fig Pairwise correlation of H_2_O_2_ inhibition and Fe^2+^-chelation Vs OH^●^ scavenging for *C*. *bonplandianus* represented in section **(A)** &**(B)**, respectively. All data are expressed as mean ± S.D. (n = 6). r = Pearson’s correlation coefficient, r^2^ = coefficient of determination and *P* = significance value.
(TIF)

S2 Fig Photomicrographs (400×) of the histopathological examinations of the liver samples of CCl_4_ group showing fibrosis. (TIF)

S3 Fig Molecular docking image. Molecular docking (molecular surface view) between NFκβ protein and Campesterol.
(TIF)

S1 Table IC_50_ values of *Croton bonplandianus* (CBL) and standard for different antioxidant and free radical scavenging assays. (DOC)

S2 Table Describe the effect of CBL on liver histology parameters of the CCl_4_ induced injured liver. (DOC)

S3 Table Chemical fingerprint of CBL extract revealed by GC-MS analyses. (DOCX)

## References

[1] Di Meo S, Reed TT, Venditti P, Victor VM. Role of ROS and RNS sources in physiological and pathological conditions. Oxid Med Cell Longev. 2016;12: 1–44. 10.1155/2016/1245049 PMC4960346 27478531

[2] Dey P, Dutta S, Biswas-Raha A, Sarkar MP, Chaudhuri TK. Haloalkane induced hepatic insult in murine model: amelioration by Oleander through antioxidant and anti-inflammatory activities, an in vitro and in vivo study. BMC Complement Altern Med. 2016;16:280–95. doi: 10.1186/s12906-016-1260-4 27516209 10.1186/s12906-016-1260-4 PMC4982413

[3] Apel K, Hirt H. Reactive oxygen species: Metabolism, oxidative stress, and signal transduction. Annu Rev Plant Biol. 2004;55:373–99. doi: 10.1146/annurev.arplant.55.031903.141701 15377225 10.1146/annurev.arplant.55.031903.141701

[4] Barzegar A, Moosavi-Movahedi AA. Intracellular ROS protection efficiency and free radical-scavenging activity of curcumin. PLoS One. 2011;6:e26012 doi: 10.1371/journal.pone.0026012 22016801 10.1371/journal.pone.0026012 PMC3189944

[5] De Iuliis GN, Newey RJ, King BV, Aitken RJ. Mobile phone radiation induces reactive oxygen species production and DNA damage in human spermatozoa in vitro. PloS one. 2009;4:e6446 doi: 10.1371/journal.pone.0006446 19649291 10.1371/journal.pone.0006446 PMC2714176

[6] Mittler R. Oxidative stress, antioxidants and stress tolerance. Trends Plant Sci. 2002;7:405–10. 12234732 10.1016/s1360-1385(02)02312-9

[7] Cichoż-Lach H, Michalak A. Oxidative stress as a crucial factor in liver diseases. World J Gastroenterol. 2014;20:8082–91. doi: 10.3748/wjg.v20.i25.8082 25009380 10.3748/wjg.v20.i25.8082 PMC4081679

[8] McCord JM. The evolution of free radicals and oxidative stress. The American J Med. 2000;108:652–59. 10.1016/s0002-9343(00)00412-5 10856414

[9] Cederbaum AI, Lu Y, Wu D. Role of oxidative stress in alcohol-induced liver injury. Arch Toxicol. 2009;83:519–48. doi: 10.1007/s00204-009-0432-0 19448996 10.1007/s00204-009-0432-0

[10] Dey P, Dutta S, Sarkar MP, Chaudhuri TK. Assesment of hepatoprotective potential of *N*. *indicum* leaf on haloalkane xenobiotic induced liver injury in swiss albino mice. Chem Biol Interact. 2015;235:37–46. doi: 10.1016/j.cbi.2015.03.025 25871905 10.1016/j.cbi.2015.03.025

[11] Vadlapudi V. *In vitro* antimicrobial activity of methanolic extract of selected indian medicinal plants. Pharmacophore. 2010;1(3):214–19.

[12] Nishanta R, Harris CS, Towers GHN. Antimicro-bial activity of plants collected from serpentine outcrops in Sri Lanka. Pharm Biol. 2012;40(3):235–44.

[13] Islam MS, Rahman MM, Rahman MA, Qayum MA, Alam MF. *In vitro* evaluation of *Croton Bonplandianum* Baill. as potential antitumor properties using *Agrobacterium tumefaciens*. J Agr Technol. 2010;6:79–86.

[14] Chaudhuri AB. Endangered medicinal plants. Delhi: Daya publishing House, 2007, pp. 226.

[15] Bhakat RK, Sen UK. Ethno medicinal plant conservation through sacred groves. Tribes and Tribals. 2008;2:55–8.

[16] Maria CMT, Joao CA, Gilvendete MPS, Manoel AN, Edilberto RS, Leticia VCL, Danie PB, Jose DBMF, Francisco AV, Otilia DLP. Larvicidal and nematicidal Activities of the leaf essential oil of *Croton regelianus*. J Chem Biodiv. 2008;5(12):2724–728. 10.1002/cbdv.200890227 19089831

[17] Hazra B, Sarkar R, Biswas S, Mandal N. Comparative study of the antioxidant and reactive oxygen species scavenging properties in the extracts of the fruits of *Terminalia chebula*, *Terminalia belerica* and *Emblica officinalis*. BMC Complement Altern Med. 2010;10:20 doi: 10.1186/1472-6882-10-20 20462461 10.1186/1472-6882-10-20 PMC2887379

[18] Mahakunakorn P, Tohda M, Murakami Y, Matsumoto K, Watanabe H. Antioxidant and free radical-scavenging activity of Choto-san and its related constituents. Biol Pharm Bull. 2004;27:38–46. 14709896 10.1248/bpb.27.38

[19] Dey P, Dutta S, Chaudhuri TK. Evaluation of erythrocyte membrane stabilizing activity, haemolytic activity and cytotoxic effect of the areal tubers of *Dioscorea alata*of north-eastern region of India. J Pharmaceu Sci Inno. 2013;2:1–4.

[20] Hoffman A, Spetner LM, Burke M. Ramifications of a redox switch within a normal cell: its absence in a cancer cell. Free Radi Biol Med. 2008;45:265–68. 10.1016/j.freeradbiomed.2008.03.025 18466777

[21] Marvibaigi M, Amini N, Supriyanto E, Majid FA, Jaganathan SK, Jamil S, Almaki JH, Nasiri R. Antioxidant activity and ROS-dependent apoptotic effect of *Scurrula ferruginea* (Jack) danser methanol extract in human breast cancer cell MDA-MB-231. PloS One. 2016;11:e0158942 doi: 10.1371/journal.pone.0158942 27410459 10.1371/journal.pone.0158942 PMC4943642

[22] Sadasivam S, Manickam A. Biochemical methods. India: New age international; 2008.

[23] Luck H, Bergmeyer HU. Catalase in Methods of Enzymatic Analysis. Academic Press, NewYork, NY, USA, 1971.

[24] Ellman GC. Tissue sulfhydryl groups. Arch Biochem Biophys. 1959;82:70–7. 13650640 10.1016/0003-9861(59)90090-6

[25] Chia R, Tattum MH, Jones S, Collinge J, Fisher EM, Jackson GS. Superoxide dismutase 1 and tgSOD1G93A mouse spinal cord seed fibrils, suggesting a propagative cell death mechanism in amyotrophic lateral sclerosis. PloS one. 2010;5:e10627 doi: 10.1371/journal.pone.0010627 20498711 10.1371/journal.pone.0010627 PMC2869360

[26] Freshney R. Culture of animal cells: a manual of basic technique. USA: John Wiley & Sons, Inc, 2005.

[27] Mishra S, Sahoo S, Rout KK, Nayak SK, Mishra SK, Panda PK. Hepatoprotective effect of *Barringtonia acutangula* Linn. leaves on carbon tetrachlorideinduced acute liver damage in rats. Indian J Nat Prod Resour. 2011;2:515–19.

[28] Hibbs JB, Taintor RR, Vavrin Z, Rachlin EM. Nitric oxide: a cytotoxic activated macrophage effector molecule. Bhiochem Biophys Res Commun. 1988;157:87–94. 10.1016/s0006-291x(88)80015-9 3196352

[29] Knodell RG, Ishak KG, black WC, Chen TS, Craig R, Kaplowitz N. Formulation and application of a numerical scoring system for assessing histological activity asymptomatic chronic active hepatitis. Hepatol. 1981;1:431–35. 10.1002/hep.1840010511 7308988

[30] Ruwart MJ, Wilkinson KF, Rush BD, Vidmar TJ, Peters KM, Henley KS, Appelman HD, Kim KY, Schuppan D, Hahn EG. The integrated value of serum procollagen III peptide over time predict hepatic hydroxyproline content and stainable collagen in a model of dietary cirrhosis in the rat. Hepatol. 1989;10: 801–6. 10.1002/hep.1840100509 2807158

[31] Trott O, Olson AJ. AutoDock Vina: improving the speed and accuracy of docking with a new scoring function, efficient optimization, and multithreading. J Comput Chem. 2010;31:455–61. doi: 10.1002/jcc.21334 19499576 10.1002/jcc.21334 PMC3041641

[32] Aruoma OI. Methodological consideration for characterization for potential antioxidant actions of bioactive components in plants foods. Mutat Res. 2003;532:9–20. 10.1016/s0027-5107(02)00317-2 12628499

[33] Huang D, Ou B, Prio RL. The chemistry behind antioxidant capacity assays. J Agric Food Chem. 2005;53:1841–56. doi: 10.1021/jf030723c 15769103 10.1021/jf030723c

[34] Ruprah M, Mant TGK, Flanagan RJ. Acute carbon tetrachloride poisoning in 19 patients: implications for diagnosis and treatment. Lancet. 1985;1:1027–29. 2859473 10.1016/s0140-6736(85)91624-1

[35] Zhang H, Yu CH, Jiang YP, Peng C, He K, Tang JY, Xin HL. Protective effects of polydatin from Polygonum cuspidatum against carbon tetrachloride-induced liver injury in mice. PLoS One. 2012;7:e46574 doi: 10.1371/journal.pone.0046574 23029551 10.1371/journal.pone.0046574 PMC3461010

[36] Zhang D, Jiang M, Miao D. Transplanted human amniotic membrane-derived mesenchymal stem cells ameliorate carbon tetrachloride-induced liver cirrhosis in mouse. PloS one. 2011;6:e16789 doi: 10.1371/journal.pone.0016789 21326862 10.1371/journal.pone.0016789 PMC3033905

[37] U.S. Environmental Protection Agency. Toxicological review of carbon tetrachloride; 2010. http://www.epa.gov/iris/toxreviews/0020tr.pdf. Accessed: 12/06/2015.

[38] Ohno Y, Gallin JI. Diffusion of extracellular hydrogen peroxide into intracellular compartments of human neutrophils. Studies utilizing the inactivation of myeloperoxidase by hydrogen peroxide and azide. J Biol Chem. 1985;260(14):8438–46. 2989289

[39] De Groot H, Littauer A, Hugo-Wissemann D, Wissemann P, Noll T. Lipid peroxidation and cell viability in isolated hepatocytes in a redesigned oxystat system: Evaluation of the hypothesis that lipid peroxidation, preferentially induced at low oxygen partial pressure, is decisive for CC1_4_ liver cell injury. Arch Biochem Biophys. 1988;264:591–99. 3401014 10.1016/0003-9861(88)90325-6

[40] Masuda Y, Nakamura Y. Effects of oxygen deficiency and calcium omission on carbon tetrachloride hepatotoxicity in isolated perfused livers from phenobarbital-pretreated rats. Biochem Pharmacol. 1990;40:1865–76. 2242020 10.1016/0006-2952(90)90368-u

[41] Kiezcka H, Kappus H. Oxygen dependence of CCl_4_-induced lipid peroxidation *in vitro* and *in vivo*. Toxicol Lett. 1980;5:191–96. 7466845 10.1016/0378-4274(80)90058-2

[42] Feng Y, Wang N, Ye X, Li H, Feng Y, Cheung F, Nagamatsu T. Hepatoprotective effect and its possible mechanism of Coptidis rhizoma aqueous extract on carbon tetrachlorideinduced chronic liver hepatotoxicity in rats. J Ethnopharmacol. 2011;138:683–90. doi: 10.1016/j.jep.2011.09.032 21963555 10.1016/j.jep.2011.09.032

[43] Amacher DE. A toxicologist’s guide to biomarkers of hepatic response. Hum Exp Toxicol. 2002;21:253–62. doi: 10.1191/0960327102ht247oa 12141396 10.1191/0960327102ht247oa

[44] Zimmerman HJ. Drug-induced hepatic disease, In: Plaa GL, Hewitt WR. (Eds.), Toxicology of the liver. Washington, Taylor and Francis; 1998, pp. 3–60.

[45] Zimmerman HJ. Drug-induced liver disease. Clin Liver Dis. 2000;4:73–96. 11232192 10.1016/s1089-3261(05)70097-0

[46] Weber LW, Boll M, Stampfl A. Hepatotoxicity and mechanism of action of haloalkanes: carbon tetrachloride as a toxicological model. Crit Rev Toxicol. 2003;33:105–36. doi: 10.1080/713611034 12708612 10.1080/713611034

[47] Tsai CF, Hsu YW, Chen WK, Chang WH, Yen CC, Ho YC, Lu FJ. Hepatoprotective effect of electrolyzed reduced water against carbon tetrachloride induced liver damage in mice. Food Chem Toxicol. 2009;27:2031–36. 10.1016/j.fct.2009.05.021 19477216

[48] Stohs SJ, Bagchi D. Oxidative mechanisms in the toxicity of metal-ions. Free Radical Bio Med. 1995;18:321–36. 7744317 10.1016/0891-5849(94)00159-h

[49] Flora SJ, Mittal M, Mehta A. Heavy metal induced oxidative stress and its possible reversal by chelation therapy. Indian J Med Res. 2008;128:501–23. 19106443

[50] Valko M, Izakovic M, Mazur M, Rhodes CJ, Telser J. Role of oxygen radicals in DNA damage and cancer incidence. Mol Cell Biochem. 2004;266:37–56. 15646026 10.1023/b:mcbi.0000049134.69131.89

[51] Morio LA, Chiu H, Sprowles KA, Zhou P, Heck DE, Gordon MK, Laskin DL., Distinct roles of tumor necrosis factor-alpha and nitric oxide in acute liver injury induced by carbon tetrachloride in mice. Toxicol Appl Pharmacol. 2001;172:44–51. doi: 10.1006/taap.2000.9133 11264022 10.1006/taap.2000.9133

[52] Gabele E, Froh M, Arteel GE, Uesugi T, Hellerbrand C, Scholmerich J. TNF-α is required for cholestasis-induced liver fibrosis in the mouse. Biochem Biophys Res Commun. 2009;378:348–53. doi: 10.1016/j.bbrc.2008.10.155 18996089 10.1016/j.bbrc.2008.10.155 PMC5052129

[53] Al-Shabanah OA, Alam K, Nagi MN, Al-Rikabi AC, Al-Bekairi AM. Protective effect of aminoguanidine, a nitric oxide synthase inhibitor, against carbon tetrachloride induced hepatotoxicity in mice. Life Sci. 2000;66:265–70. 10666002 10.1016/s0024-3205(99)00589-5

[54] Matsumaru K, Ji C, Kaplowitz N. Mechanisms for sensitization to TNF-induced apoptosis by acute glutathione depletion in murine hepatocytes, Hepatol. 2003;37(6): 1425–34. 10.1053/jhep.2003.50230 12774022

[55] Nagai H, Matsumaru K, Feng G, Kaplowitz N. Reduced glutathione depletion causes necrosis and sensitization to tumor necrosis factor-alphainduced apoptosis in cultured mouse hepatocytes. Hepatol. 2002;36(1):55–64. 10.1053/jhep.2002.33995 12085349

[56] Branda M, Wands JR. Signal transduction cascades and hepatitis B and C related hepatocellular carcinoma. Hepatol. 2006;43:891–902. 10.1002/hep.21196 16628664

