# Supplementary figures and images for: Amelioration of CCl4 induced liver injury in swiss albino mice by antioxidant rich leaf extract of Croton bonplandianus Baill
Source: PLoS One. 2018 Apr 30;13(4):e0196411. doi: 10.1371/journal.pone.0196411 (PMC5927454; doi:10.1371/journal.pone.0196411)

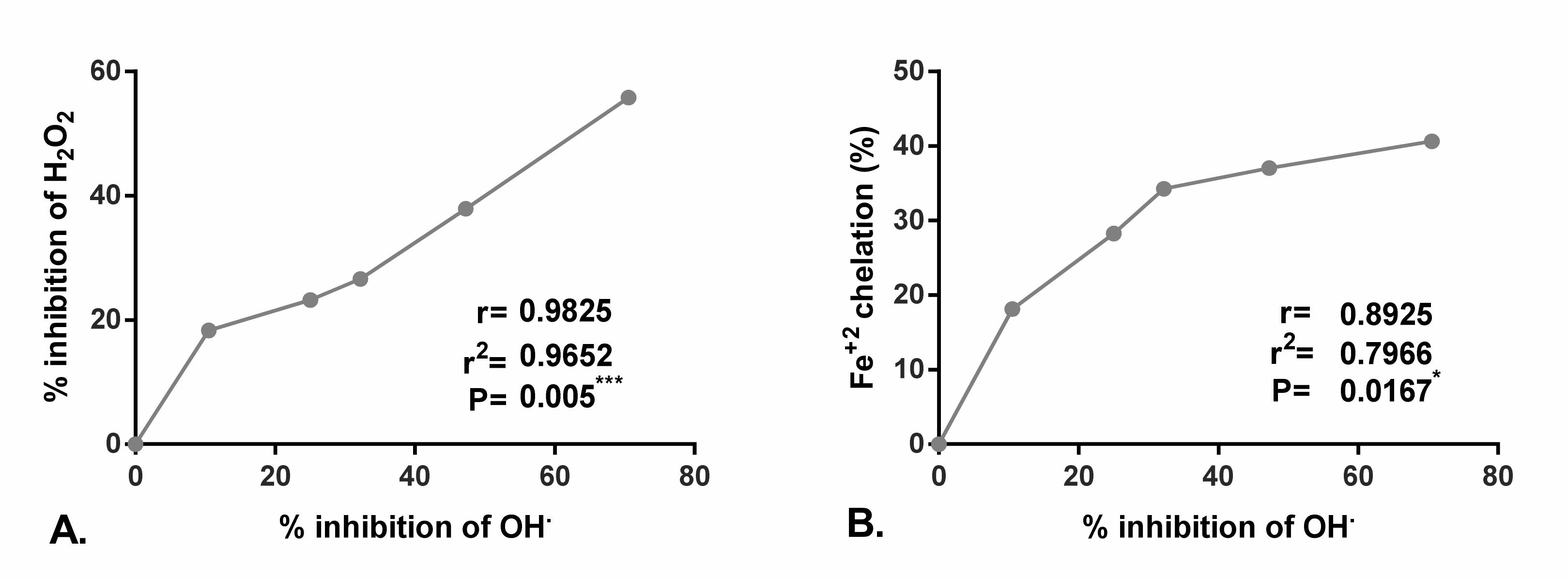

Supplement: S1 Fig — Pairwise correlation of H2O2 inhibition and Fe2+-chelation Vs OH● scavenging for C. bonplandianus represented in section (A) &(B), respectively. All data are expressed as mean ± S.D. (n = 6). r = Pearson’s correlation coefficient, r2 = coefficient of determination and P = significance value. (TIF) [file pone.0196411.s001.tif]

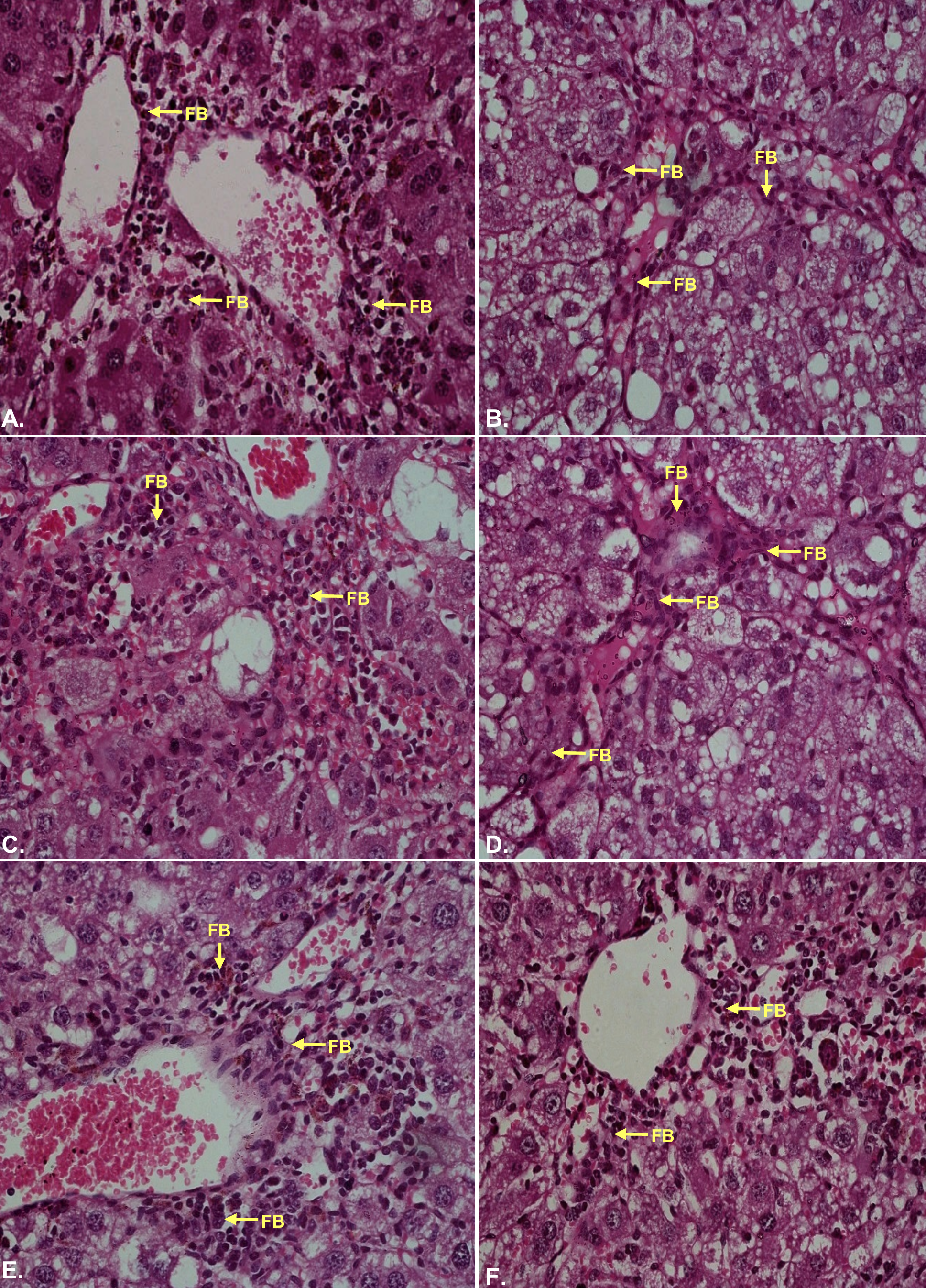

Supplement: S2 Fig — (TIF) [file pone.0196411.s002.tif]

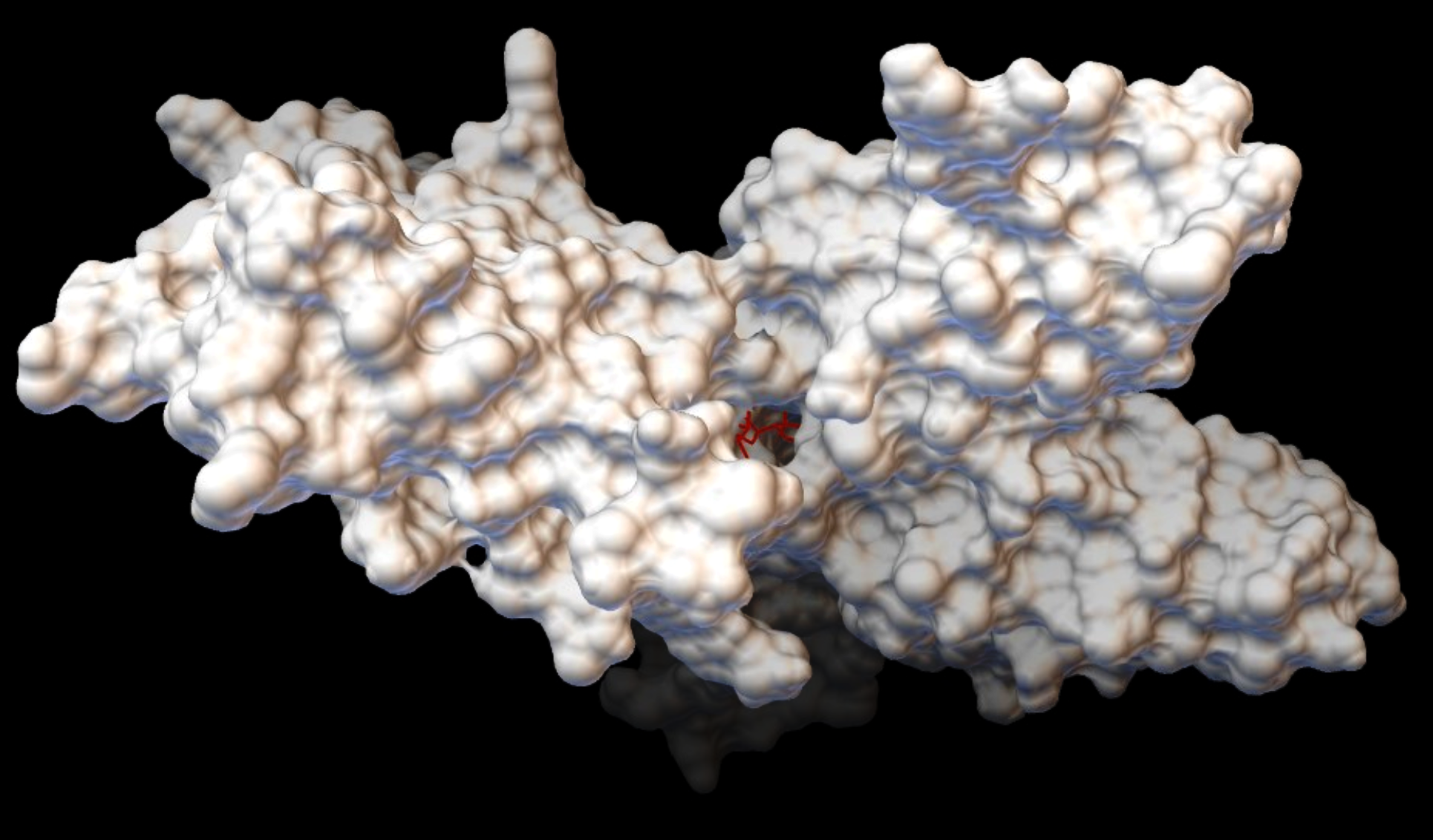

Supplement: S3 Fig — Molecular docking (molecular surface view) between NFκβ protein and Campesterol. (TIF) [file pone.0196411.s003.tif]
