# Supplementary material for: Amelioration of CCl4 induced liver injury in swiss albino mice by antioxidant rich leaf extract of Croton bonplandianus Baill
Source: PLoS One. 2018 Apr 30;13(4):e0196411. doi: 10.1371/journal.pone.0196411 (PMC5927454; doi:10.1371/journal.pone.0196411)
Supplement: S1 Table — (DOC) [file pone.0196411.s004.doc]

| **Antioxidant Parameters** | **CBL** | **Standard** |
| --- | --- | --- |
| DPPH | 3.79±0.06** | 10.49±0.77  (Ascorbic Acid) |
| Hydroxyl Radical | 173.65±4.96*** | 597.15±11.90  (Mannitol) |
| Hydrogen Peroxide | 222.95±11.53** | 2185.22±187.45  (Sodium Pyruvate) |
| Nitric Oxide | 36.74±2.79*** | 129.82±7.31  (Curcumin) |
| Superoxide Anion | 44.69±1.90*** | 94.59±3.75  (Quercetin) |
| Hypochlorous Acid | 66.58±4.39** | 117.50±10.02  (Ascorbic Acid) |
| Total Antioxidant Activity | 46.57±2.19** | 116.46±5.91  (Ascorbic Acid) |
| Peroxynitrite | 785.48±59.32**^NS^** | 785.84±59.75  (Gallic Acid) |
| Singlet Oxygen | 257.00±3.22*** | 48.41±2.02  (Lipoic Acid) |
| Lipid Peroxidation | 19.70±1.32** | 11.16±0.26  (Trolox) |
| Iron Chelation | 123.46±1.92*** | 10.23±0.89  (EDTA) |
| Units in μg/ml. Data expressed as mean ± S.D (n=6). *p<0.05; **p<0.01; ***p<0.001; NS – Non significant when compared with standard. | | |
