# Supplementary material for: Amelioration of CCl4 induced liver injury in swiss albino mice by antioxidant rich leaf extract of Croton bonplandianus Baill
Source: PLoS One. 2018 Apr 30;13(4):e0196411. doi: 10.1371/journal.pone.0196411 (PMC5927454; doi:10.1371/journal.pone.0196411)
Supplement: S2 Table — (DOC) [file pone.0196411.s005.doc]

| **Parameters studied** | **Control** | **CCl_4_** | **Silymarin** | **CBLL** | **CBLM** | **CBLH** |
| --- | --- | --- | --- | --- | --- | --- |
| Hepatocellular necrosis | 0 | 7 | 2 | 6 | 5 | 3 |
| Bile duct prolifaration | 0 | 2 | 0 | 2 | 1 | 1 |
| Sinusoidal dilatation | 0 | 1 | 0 | 1 | 0 | 0 |
| Inflammation (leukocyte infiltration) | 1 | 8 | 2 | 6 | 5 | 3 |
| Vascular congestion | 0 | 3 | 1 | 3 | 2 | 1 |
| Loss of structure of hepatic nodules | 0 | 5 | 2 | 4 | 2 | 2 |
| Hepatocellular fibrosis | 0 | 4 | 0 | 1 | 1 | 0 |
| Fatty infiltration | 0 | 1 | 0 | 0 | 0 | 0 |
| Vacuolar degeneration | 0 | 3 | 0 | 2 | 0 | 0 |
| Calsification | 0 | 2 | 1 | 1 | 1 | 0 |
| Cumulative score | 1 | 36 | 8 | 26 | 17 | 10 |
