# Supplementary material for: Amelioration of CCl4 induced liver injury in swiss albino mice by antioxidant rich leaf extract of Croton bonplandianus Baill
Source: PLoS One. 2018 Apr 30;13(4):e0196411. doi: 10.1371/journal.pone.0196411 (PMC5927454; doi:10.1371/journal.pone.0196411)
Supplement: S3 Table — (DOCX) [file pone.0196411.s006.docx]

| **Sl no** | **Compounds name** | **Molecular weight** | **Formula** | **RT** |
| --- | --- | --- | --- | --- |
|  | 2-Pyrrolidinone, 1-methyl- | 99 | C5H9NO | 9.20 |
|  | Propanoic acid, 2-[(trimethylsilyl)oxy]-, trimethylsilyl ester | 234 | C9H22O3Si2 | 9.83 |
|  | Hexanoic acid, trimethylsilyl ester | 188 | C9H20O2Si | 10.01 |
|  | 2-Methyl-4-pentenoic acid, trimethylsilyl ester | 186 | C9H18O2Si | 10.14 |
|  | Acetic acid, [(trimethylsilyl)oxy]-, trimethylsilyl ester | 220 | C8H20O3Si2 | 10.19 |
|  | 2-Hexenoic acid, trimethylsilyl ester | 185 | C9H18O2Si | 11.26 |
|  | Ethanedioic acid, bis(trimethylsilyl) ester  (syn. Oxalic acid, bis(trimethylsilyl) ester) | 234 | C8H18O4Si2 | 11.72 |
|  | Propanoic acid, 3-[(trimethylsilyl)oxy] trimethylsilyl ester | 234 | C9H22O3Si2 | 12.01 |
|  | p-Trimethylsiloxybenzaldehydeoxime, trimethylsilyl- | 281 | C13H23NO2Si2 | 12.09 |
|  | Butanoic acid, 3-[(trimethylsilyl)oxy]-, trimethylsilyl ester (syn. β-Hydroxybutyric acid (2TMS)) | 248 | C10H24O3Si2 | 12.47 |
|  | Propanedioic acid, bis(trimethylsilyl) ester | 248 | C9H20O4Si2 | 13.66 |
|  | D-Erythro-Pentonic acid, 2-deoxy-3,5-bis-O-(trimethylsilyl)-, γ-lactone | 276 | C11H24O4Si2 | 14.13 |
|  | 1,3-Bis(trimethylsilyloxy)butane | 234 | C10H26O2Si2 | 14.46 |
|  | 1-Phenylethanol, tert-butyldimethylsilyl ether | 236 | C14H24OSi | 14.63 |
|  | Octanoic acid, trimethylsilyl ester | 216 | C11H24O2Si | 15.11 |
|  | Glycerol, tris(trimethylsilyl) ether | 308 | C12H32O3Si3 | 15.70 |
|  | Phosphoric acid, tris(trimethylsilyl) ester | 314 | C9H27O4PSi3 | 15.77 |
|  | Butane, 1,2,3-tris(trimethylsiloxy)- | 322 | C13H34O3Si3 | 16.19 |
|  | Butanedioic acid, bis(trimethylsilyl) ester | 262 | C10H22O4Si2 | 16.55 |
|  | Glyceric acid, (3TMS) | 322 | C12H30O4Si3 | 17.20 |
|  | Itaconic acid, bis-TMS ester | 274 | C11H22O4Si2 | 17.35 |
|  | Fumaric acid, bis(trimethylsilyl) ester | 260 | C10H20O4Si2 | 17.40 |
|  | Methylmaleic acid, bis(trimethylsilyl) ester | 274 | C11H22O4Si2 | 17.57 |
|  | 2(3H)-Furanone, dihydro-3,4-is[(trimethylsilyl)oxy]-, trans- | 262 | C10H22O4Si2 | 18.19 |
|  | Methylmaleic acid, bis(trimethylsilyl) ester | 274 | C11H22O4Si2 | 18.78 |
|  | β-Caryophyllen | 204 | C15H24 | 19.25 |
|  | Decanoic acid, trimethylsilyl ester | 244 | C13H28O2Si | 20.11 |
|  | Butanedioic acid, [(trimethylsilyl)oxy]-, bis(trimethylsilyl) ester (syn. Malic acid (3TMS) | 350 | C13H30O5Si3 | 21.24 |
|  | L-Threonic acid, tris(trimethylsilyl) ether, trimethylsilyl ester | 424 | C16H40O5Si4 | 23.17 |
|  | Pentanedioic acid, 2-[(trimethylsilyl)oxy]-, bis(trimethylsilyl) ester (syn. 2-Hydroxyglutaric acid, tri-TMS) | 364 | C14H32O5Si3 | 23.26 |
|  | Ethanol, 2-(octadecyloxy)-(syn. 2-Octadecyloxyethanol) | 314 | C20H42O2 | 23.44 |
|  | Dodecanoic acid, trimethylsilyl ester | 272 | C15H32O2Si | 24.71 |
|  | Ribitol, 1,2,3,4,5-pentakis-O-(trimethylsilyl)- | 512 | C20H52O5Si5 | 25.17 |
|  | 14-Methyl-pentadecane-1,2-diol, bis(trimethylsilyl) ether | 402 | C22H50O2Si2 | 26.57 |
|  | Benzoic acid, 2-[(trimethylsilyl)oxy]-, trimethylsilyl ester(syn. Benzoic acid, 2-[(trimethylsilyl)oxy]-, trimethylsilyl ester) | 282 | C13H22O3Si2 | 26.76 |
|  | 5,8,11-Eicosatriynoic acid, trimethylsilyl ester | 372 | C23H36O2Si | 26.97 |
|  | Ethanol, (2-(3,4-dihydroxyphenyl)-, tris(trimethylsilyl)- | 370 | C17H34O3Si3 | 27.43 |
|  | Cinnamic acid, p-(trimethylsiloxy)-, trimethylsilyl ester (syn. p-Coumaric acid, bis-TMS) | 308 | C15H24O3Si2 | 27.79 |
|  | Tetradecanoic acid, trimethylsilyl ester | 300 | C17H36O2Si | 28.90 |
|  | D-Pinitol, pentakis(trimethylsilyl) ether | 554 | C22H54O6Si5 | 29.87 |
|  | 1,5-Anhydro-D-sorbitol, tetrakis(trimethylsilyl) ether | 452 | C18H44O5Si4 | 30.06 |
|  | n-Hexadecanoic acid (syn. Palmitic acid) | 256 | C16H32O2 | 31.13 |
|  | L-Ascorbic acid, 2,3,5,6-tetrakis-O-(trimethylsilyl)-(syn. Ascorbic acid (4TMS)) | 464 | C18H40O6Si4 | 31.42 |
|  | Hexadecanoic acid, trimethylsilyl ester | 328 | C19H40O2Si | 32.75 |
|  | Phytol | 296 | C20H40O | 33.95 |
|  |  |  |  |  |
|  | 9,12-Octadecadienoic acid (Z,Z)- (syn. Linoleic acid) | 280 | C18H32O2 | 34.28 |
|  | 9,12,15-Octadecatrienoic acid, (Z,Z,Z)- | 278 | C18H30O2 | 34.41 |
|  | Octadecanoic acid (stn. Stearic acid,) | 284 | C18H36O2 | 34.81 |
|  | Ethyl iso-allocholate | 436 | C26H44O5 | 35.28 |
|  | α-Linolenic acid, trimethylsilyl ester | 350 | C21H38O2Si | 35.83 |
|  | Octadecanoic acid, trimethylsilyl ester  (syn. Stearic acid, trimethylsilyl ester) | 356 | C21H44O2Si | 36.26 |
|  | Eicosanoic acid, trimethylsilyl ester(syn. Arachidic acid, trimethylsilyl ester) | 384 | C23H48O2Si | 29.50 |
|  | psi.,.psi.-Carotene, 1,1',2,2'-tetrahydro-1,1'-dimethoxy- | 600 | C42H64O2 | 40.00 |
|  | Sucrose, octakis(trimethylsilyl) ether | 918 | C36H86O11Si8 | 41.70 |
|  | D-(+)-Turanose, octakis(trimethylsilyl) ether | 918 | C36H86O11Si8 | 42.04 |
|  | α-D-Glucopyranoside, 1,3,4,6-tetrakis-O-(trimethylsilyl)-β-D-fructofuranosyl 2,3,4,6-tetrakis-O-(trimethylsilyl)- | 918 | C36H86O11Si8 | 42.66 |
|  | 1-Monolinoleoylglycerol trimethylsilyl ether | 498 | C27H54O4Si2 | 44.49 |
|  | Squalene | 410 | C30H50 | 45.20 |
|  | Hentriacontane | 436 | C31H64 | 46.10 |
|  | Tocopherol-γ-tms-derivative | 488 | C31H56O2Si | 47.62 |
|  | 1-Octacosanol, trimethylsilyl ether | 482 | C31H66OSi | 49.29 |
|  | (+)-α-Tocopherol, O-trimethylsilyl- | 502 | C32H58O2Si | 49.46 |
|  | 17-Pentatriacontene | 490 | C35H70 | 50.11 |
|  | Campesterol, TMS ether | 472 | C31H56OSi | 50.95 |
|  | Stigmasterol trimethylsilyl ether | 484 | C32H56OSi | 51.38 |
|  | β-Sitosteroltrimethylsilyl ether | 586 | C32H58OSi | 52.23 |
|  | α-Amyrin, trimethylsilyl ether | 498 | C33H58OSi | 52.98 |
